# Advances in Cell-Conductive Polymer Biointerfaces and Role of the Plasma Membrane

**DOI:** 10.1021/acs.chemrev.1c00363

**Published:** 2021-09-28

**Authors:** Anna Mariano, Claudia Lubrano, Ugo Bruno, Chiara Ausilio, Nikita Bhupesh Dinger, Francesca Santoro

**Affiliations:** † Tissue Electronics, Istituto Italiano di Tecnologia, 80125 Naples, Italy; ‡ Dipartimento di Chimica, Materiali e Produzione Industriale, Università di Napoli Federico II, 80125 Naples, Italy

## Abstract

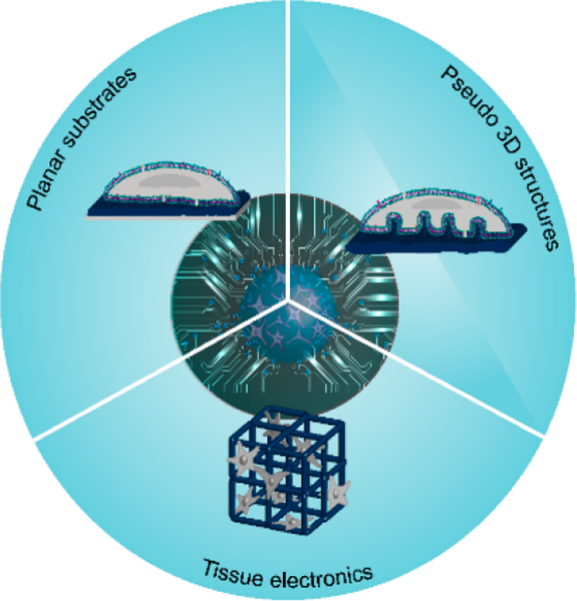

The plasma membrane (PM) is often described as a wall, a physical barrier separating the cell cytoplasm from the extracellular matrix (ECM). Yet, this wall is a highly dynamic structure that can stretch, bend, and bud, allowing cells to respond and adapt to their surrounding environment. Inspired by shapes and geometries found in the biological world and exploiting the intrinsic properties of conductive polymers (CPs), several biomimetic strategies based on substrate dimensionality have been tailored in order to optimize the cell–chip coupling. Furthermore, device biofunctionalization through the use of ECM proteins or lipid bilayers have proven successful approaches to further maximize interfacial interactions. As the bio-electronic field aims at narrowing the gap between the electronic and the biological world, the possibility of effectively disguising conductive materials to “trick” cells to recognize artificial devices as part of their biological environment is a promising approach on the road to the seamless platform integration with cells.

## 1 Introduction

The plasma membrane (PM) constitutes the fundamental border between the cell and its extracellular environment, tightly coordinating several processes as the cell interfaces with the extracellular matrix (ECM).

Cells are in fact embedded in an intricate meshwork of proteins, proteoglycans, and glycosaminoglycans constituting the ECM: the composition, concentration, and cross-linking structures forming this web, determining matrix stiffness as well as creating pseudo-3D micro- and nanoscale features, present cells with tissue-specific biochemical, mechanical, and topographical cues.^1−5^ As the cell first outpost at the boundary with the extracellular world, the PM enables the cell to sense and respond to environmental changes, playing a crucial role in regulating cell homeostasis and function.^4,6^ The PM-mediated cross-talk between the cell and its surroundings is critical in bio-electronics, where the understanding of the processes at the cell–material interface that regulate cell adhesion and behavior becomes a necessary requirement for the efficient development of bio-electronic platforms and their perfect integration within tissues.^7^ Historically, conductive inorganic materials such as metals and silicon have played a major role in the development of electronic devices.^8^ However, despite inorganic materials displaying many biologically relevant electrical and physical properties, the mechanical mismatch between the hard electronics and the much softer biological matter may hinder their long-lasting communication with cells and tissues.^8^

In this scenario, conducting polymers (CPs), as soft materials exhibiting both ionic and electronic conduction, have come to play a major role in bio-electronic applications, matching both mechanical and conduction properties of living systems.^9−12^ Importantly, the intrinsic ability of such materials to convert an ion flow to different electronic conduction states guarantees straightforward and high-efficiency signal transduction at the interface, surpassing de facto another important limitation of the inorganic materials, which are not permeable to ions.^13^

As the bio-electronics field chases its fundamental aspiration to overcome the gap between the electronic and the biological world, several mimicry strategies have been developed, in order to effectively disguise conductive materials to “trick” cells to recognize artificial devices as part of their biological environment, thus maximizing cellular interactions at the interface.^14^

In this review we highlight how the great effort deployed to unwind intracellular and extracellular dynamics has opened up the possibility to engineer conductive materials such as CPs, which, just like the ECM might provide the chemical, topographical, and mechanical support necessary to promote cell–substrate and cell–cell interactions.^15^ These strategies can be further exploited to trigger desired cellular behaviors at the interface, including cell proliferation, differentiation, as well as promoting tissue generation.^15^

**Figure 1:**
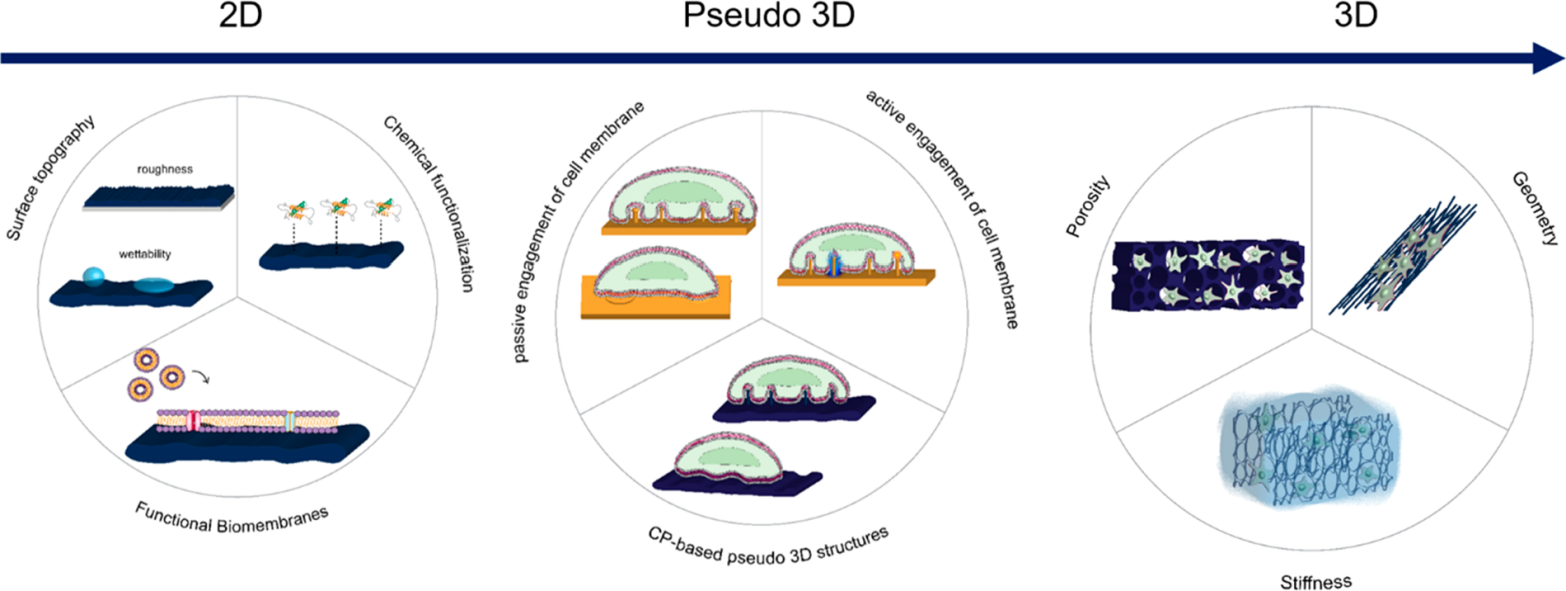
Schematic overview of this review, depicting biomimetic strategies to achieve tight cell–chip coupling, transitioning from planar to 3D CP-based materials.

Here, we discuss how biomimetic strategies on planar, pseudo-3D and 3D CP-based substrates can be tailored to achieve a tight engagement with the cell PM and improve coupling, as shown in Figure 1.

More in detail, we present substrate roughness and chemical functionalization as a powerful tool to improve mimicry strategies on planar CPs. The presence of adhesive ECM proteins or growth factors, either adsorbed or covalently bound to the surface of CPs, provide chemical cues to cells, stimulating adhesion and providing control over cell functions.^16^ Furthermore, we suggest the use of supported lipid bilayers (SLBs) as a promising biomimetic approach to improve cell–chip coupling.

We also underline how engineering strategies based on the substrate dimensionality have enabled the development of more effective bio-interfaces. In particular, the shift from planar to pseudo-3D inorganic electrodes and the possibility of exploiting the cell’s ability to respond to local changes in topography have significantly enhanced the electrical coupling at the interface, leading to improved electrophysiological recordings and the detection of cell subthreshold electrical activity.^17^ We also discuss how inorganic pseudo-3D substrates have not only been instrumental in deepening our understanding of the interfacial interactions governing the cell–material interface, but also in inspiring pioneering works aimed at the development of CP-based pseudo-3D bio-interfaces.

Lastly, we highlight how conductive 3D tissue-like platforms, recapitulating the complex 3D ECM architecture and further improving the cell–substrate interactions, can successfully trigger specific cellular responses at the cell–material interface.^15,18,19^ In conclusion, engineering biomimetic electronics appears a very promising approach in order to gain an intimate coupling between artificial electronic devices and cells, paving the way toward a new class of in vitro platforms seamlessly integrated within living tissues.

## 2 Cell–Chip Coupling in 2D Systems

### 2.1 Cell–Surface Electrode Interactions and Modeling in 2D Systems

**Figure 2:**
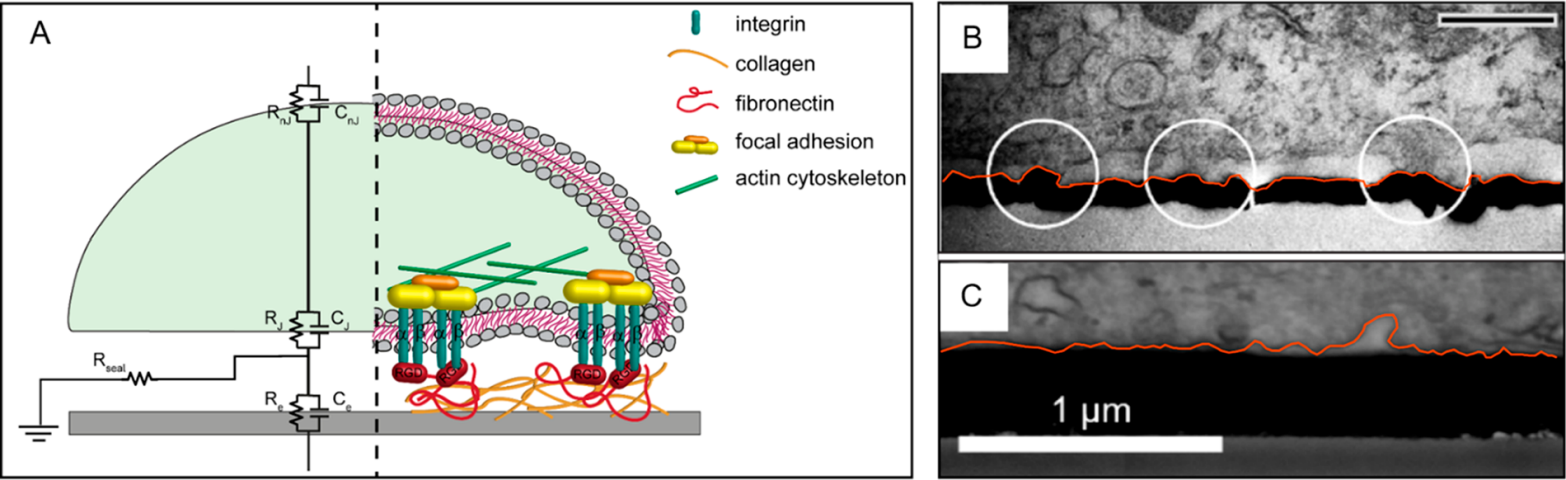
Cell–electrode interactions in 2D systems. (A) On the left, electrical equivalent circuit modeling of the cell–electrode interface; on the right, schematic representation of the integrin-mediated cytoskeletal activation and focal adhesion proteins maturation at the contact area between a cell and a planar electrode. (B) Transmission electron micrograph showing sites of PM ruffling and tight adhesion areas (white circles). Scale bar: 1 μm. (C) Focused ion beam–scanning electron micrograph showing the PM and local ruffling on a planar PEDOT substrate. Orange line indicates the PM profile. Reprinted and adapted with permission from the following: Ref (28). Copyright 2007 The Royal Society. Ref (29). Copyright 2017 American Chemical Society.

The coupling between electrogenic cells and external devices is essential for cell recordings and stimulation. Therefore, deepening our understanding of the interactions between cells and artificial materials, to develop effective interfaces, becomes of critical importance. In general, cell adhesion and spreading onto planar materials, thus electrodes, involves the activation of transmembrane receptors, known as integrins (Figure 2A).^4,20^ Integrins are mechanosensitive: they do not only respond to the biochemical composition of their environment (e.g., collagen, fibronectin, and so on) as shown in Figure 2A but may also respond to the biophysical properties of a planar substrate (e.g., stiffness).^21,22^

Upon activation, integrins cluster together and, triggering the assembly of adaptor, scaffold, and signaling proteins, induce the formation of focal adhesion sites at the cell–substrate interface,^23−26^ as depicted in Figure 2A^20,23,26^ The physical association with the cytoskeleton, acting as a bridge between the outside and the inside of the cell^20,23,26^ and locally modulating actin organization and dynamics (e.g., nucleation, cross-linking, bundling, and actin–myosin contractility), generates distinct architectures that stabilize cell–substrate adhesion.^27^

Altogether, cytoskeletal systems are dynamic and adaptable, making the cell–substrate physical coupling a nonstatic process.^30^ In fact, mechanical interactions of focal adhesions sites, mediating the continuous reshaping and adaptation of the cell on the materials surface, can generate local traction forces on the substrate that can be used by the cell to propel directional movement by contact guidance.^31^

If the organization of the cytoskeleton is essential in promoting the maturation of anchoring points distributed through focal adhesion sites,^27^ its dynamic and continuous rearrangement is also critical for the formation of cellular protrusions such as filopodia, and filopodia-like structures, also involved in substrate tethering and sensing.^32^ Because the cell is enclosed by the PM, the formation of these protrusions is also supported by local membrane trafficking and budding, further highlighting the significant role played by the cell PM in regulating cell adhesion and spreading.^33−35^

In turn, the physical coupling between cells and planar conductive materials has a major impact on the electrical conduction mechanism at the interface, both for sensing and stimulation.^36^

In this scenario, a first model of the cell–electrode interface was proposed back in the 1990s, when Fromherz et al. described the direct electrical coupling between Retzius cells isolated from the leech *Hirudo medicinalis* and an open gate of a p-channel field-effect transistor (FET).^37^ This study paved the way for the modeling of the electrical coupling between a planar device and excitable cells: an equivalent electrical circuit was proposed and the recordings of voltages, resembling the first derivative of action potentials, were shown.^37^ Later studies highlighted that the strength of the coupling is variable and might be ascribed to an inhomogeneous contact between the cell membrane and the electrode.^38^ This phenomenon is given by the unevenness in the width of a cleft forming at the interface domain and, second, even in a homogeneous contact, the current flowing across the cleft may vary the intensity of the voltage recorded by the electrode.^38,39^

It is now widely recognized that the interface between cells and substrate-integrated planar electrodes can be described by an equivalent electrical circuit (point-contact model), in which lumped elements are exploited to depict the electrical coupling,^40^ as shown in Figure 2A. Here, the cell–electrode system is composed of three components: the electrode, the cell, and the cleft formed between the two.^17^ As the cell membrane interfaces with the planar electrode, it is possible to discriminate between two domains: a junctional membrane, facing the electrode, represented by a junctional resistance (*R*_j_) and conductance (*C*_j_) and a nonjunctional membrane domain, facing the surrounding media, represented by a nonjunctional resistance (*R*_nj_) and capacitance (*C*_nj_). The electrode is characterized by a resistance *R*_e_ and a capacitance *C*_e,_ that describe the formation of an electrical double layer (EDL) of a metal surface in an ionic buffer solution.^41^ The junctional membrane surface depends on the cell–electrode contact area and the ability of cells to flatten on a planar substrate. A cleft, filled with an ionic solution (the cell media) separates the cell PM and the electrode and thus generates a resistance that is referred to as seal resistance (*R*_seal_). The cleft width influences the amplitude of the local field generated by the cell electrical activity, thus determining the efficiency of the electrical coupling.^42^ The resulting sealing resistance affects both the amplitude and shape of field potential recordings.^43^

As a comparison, the gold standard method for intracellular recordings of excitable cells is the patch clamp technique, in which the cell membrane is suctioned into a recording pipette. This tight interface accounts for a sealing resistance in the range of 10–100 GΩ, allowing for the recording of currents flowing across the membrane.^44^

In the case of planar electrodes, several studies have shown that the cleft width ranges from 40 to 100 nm,^29,45,46^ (Figure 2B,C). This corresponds to a *R*_seal_ of 1–2 MΩ, resulting in field potential recordings in the range of a few hundreds of microvolts.^17^ Consequently, extracellular field potentials (typically in the range of 100 μV) recorded from small mammalian neurons can only be detected with devices with low noise.^47^ These circumstances make planar electrodes effectively blind to subthreshold potentials that, in the case of the neural cells, represent the majority of the electrical signaling and cover an essential role in brain functions.^48,49^

Likewise, the electrical stimulation of cells through planar electrodes is highly dependent on the coupling conditions. The size of the electrodes is crucial in the propagation of electrical stimuli: a bigger electrode accounts for a lower impedance, enhancing the stimulation.^41,50^ On the other hand, the choice of a smaller electrode increases the spatial resolution, eliciting an electrical response solely in cells in the near surroundings of the stimulation area.^51^

In this scenario, high-density multielectrode arrays (HD-MEAs) represent a possible strategy for the optimization of both stimulation and recording, in terms of temporal and spatial resolution, along with increased recordings amplitude.^52^

In addition, the use of lumped parameters in the cell–electrode electrical equivalent model often does not depict correctly all mechanisms occurring at this hybrid interface, including the highly dynamic mechanical behavior of cells. Recently, Bruno et al. proposed a mathematical model and a system theory approach to numerical simulations to describe and predict the cell–electrode interfacial interactions over time. In particular, the cleft and the portion of the cell directly in contact with the electrode surface are defined as mathematical functions of time. The transition from a parametrical to a functional modeling allows for a dynamical and more realistic description of the coupling, in which the interface may vary dynamically. This approach allows for defining and simulating specific cell mechanical displacements and adhesion processes, assessing the interface variation and enabling the optimization process of electrodes shape and size.^53^

### 2.2 Engineering the Planar Cell–Electrode Bio-interface

Optimizing cell–chip bio-interfaces in planar conditions requires fine-tuning of multiple surface properties. In particular, roughness and biofunctionalization can be engineered to recapitulate ECM topographical and chemical cues and tighten cell–chip interactions. In the following sections, we will discuss the effect of these properties on the PM, focusing on their modulation through CPs.

#### 2.2.1 Surface Topography and Wettability

Given the central role played by the ECM micro- and nanotopographies in engaging adhesive contact points with cell PM^2^, the possibility of tuning the surface roughness of planar materials becomes of critical importance when developing effective bio-interfaces.^54^ Surface modification approaches, aiming at increasing the electrode surface area effectively available to interface with cells, have been successfully used to improve the cell–chip electrical coupling with inorganic electrodes.^54−57^ For instance, rough gold and platinum black electrodes, increasing the effective contact area, showed reduced impedance allowing for increased amplitude recording.^54^ Similar modifications of planar microelectrodes with a gold nanoflake structure^58^ or with entangled carbon nanotube layers^55−57^ resulted in a dramatic reduction in impedance, enabling the acquisition of high-quality recordings and the stimulation of interfacing neurons, while providing contact guidance cues.

Alternatively, CPs can be employed as a functional coating to engineer local topographies, while showing reduced impedance in aqueous environment compared to inorganic electrodes.^59,60^ In this context, CPs thin films can be easily modified to achieve either smooth or rough surfaces by tuning the deposition parameters, i.e., controlling the speed rotation in the spin coating process^61^ or the voltage/current applied in the case of electrodeposition.^62^ The latter was used to achieve CP-coated electrodes whose fuzzy film morphology increased the electroactive surface area and caused a significant decrease of the electrodes’ impedance.^63,64^ Importantly, CPs can be precisely engineered with both micro- and nanometer scale features recapitulating the ECM architecture.^65^ For instance, Hardy et al. used a silk-polypyrrole (PPy) film with micrometer-scale β-sheet silk structures which facilitated dorsal root ganglion (DRG) cells attachment while the topographical distribution of the grooves, providing contact guidance cues, promoted the aligned extension of neurites.^65^ On the other hand, Liu et al. electropolymerized PPy nanoscaled particles with an average diameter of 62 nm in order to develop a nanostructured membrane. Here, electron microscopy investigations showed osteoblasts flattening on the nano-PPy membrane, suggesting remarkable cell adhesion. Furthermore, cells exhibited a polygonal morphology, typical of their phenotype, along with cytoplasmic extensions, suggesting the topography-driven activation of the intracellular cytoskeleton.^66^ More examples on engineered CPs used to control local surface roughness can be found in Table 1, along with further details on materials and cell types.

**Table 1:** CP-Based Surface Functionalization Approaches of Planar Inorganic Substrates

device	material	functionalization	cell type	application	ref
silicon probe	gold	PPy/PSS	ex vivo brain from guinea pig	Reduced impedance	(64)
silicon probe	gold	PEDOT/DCDPGYIGSR	rat glial cells	Reduced impedance	(63)
substrate type	glass	PEDOT:PSS/PEGDA	primary chicken fibroblasts	cell adhesion and spreading	(73)
film	silicon	PANi	PC-12 cells	cell adhesion and proliferation	(74)
film	PU	PPy nanoparticles	C2C12 cells	cell adhesion and differentiation	(75)
film	polyester fabrics	PPy	HUVEC cells	cell adhesion and proliferation	(76)

Surface roughness might also influence the polymer wettability,^67−69^ another key property of CPs that can affect the coupling with cells, even though the preference for a hydrophobic or a hydrophilic substrate might depend on the cell type.^67−69^ Both local surface roughness and wettability can be influenced by electrochemical doping/dedoping of CPs from a neutral state (dedoped) to an oxidized one (p-doped) upon the application of an external voltage.^70,71^ Exploiting this mechanism, Borin et al. engineered three-terminal organic electrochemical transistors (OECT) using poly(3,4-ethylenedioxythiophene) (PEDOT):tosylate, to create cell-adhesive and cell-repellent regions along the transistor channel through the application of a certain potential. Here, regulating the gate voltage, and therefore the degree of oxidation, it was possible to control MDCK cells adhesion and distribution in a region of interest.^72^

#### 2.2.2 Surface Chemical Functionalization

Cell attachment on planar conductive materials can be strongly promoted by surface chemical functionalization strategies.

**Figure 3:**
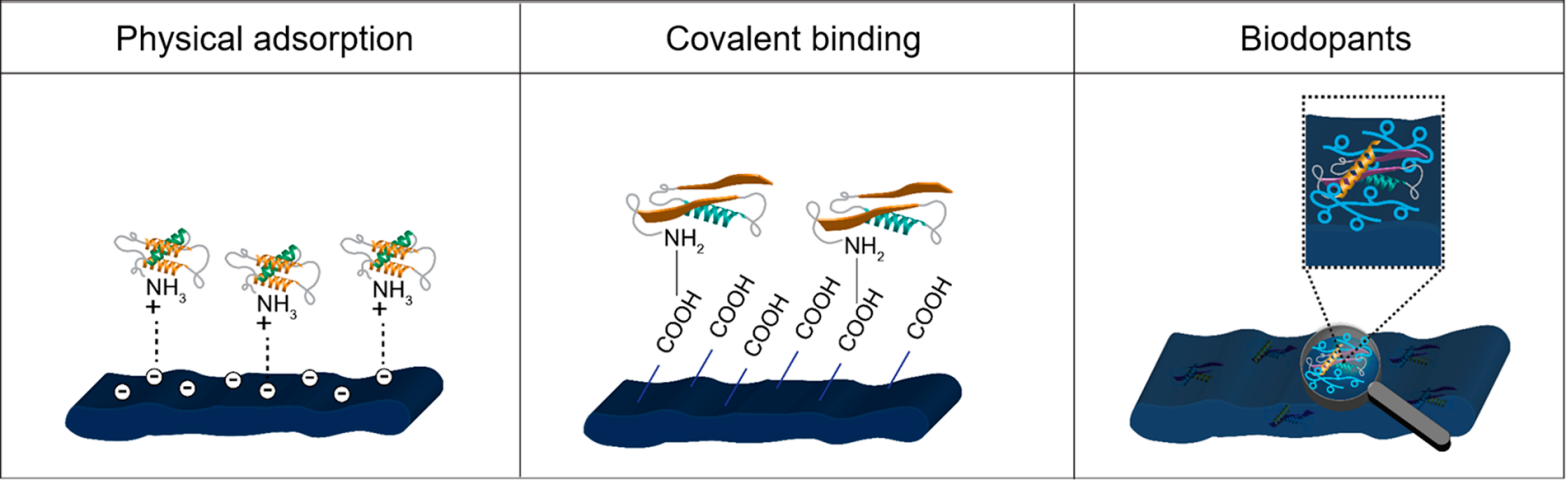
Chemical functionalization strategies. Schematic layout depicting chemical functionalization approaches: physical adsorption, covalent binding, and biodopants.

For instance, the presence of protonated lysine groups covalently immobilized on the backbone of a dithienyl-diketopyrrolopyrrole and thiophene (DPP3T), increasing the surface charge of the film, was shown to enhance primary neurons adhesion and growth.^77^ In addition, exploiting the ability of cells to selectively recognize and bind biomolecules found in native ECM, chemical functionalization of conductive materials with biomimetic cell-adhesive molecules can be used to gain a tighter apposition of PM onto planar substrates,^16^ as shown in Figure 3.

CPs indeed can undergo multiple functionalization strategies where proteins can be physically adsorbed, covalently bound on the polymer surface, or embedded within the conductive polymer matrix.^16^ Physical adsorption represents probably a straightforward approach for biofunctionalization:^78^ for example, the adsorption of nanofibrillar collagen type IV on poly(3-hexylthiophene) (P3HT) and PEDOT:poly(styrenesulfonate) (PSS) films significantly increased the number of adhering cardiomyocytes and 3T3 fibroblasts cells on otherwise poorly adhesive surfaces. Furthermore, fibroblasts cultured on these films showed a well-spread morphology when the substrates were coated with collagen type IV, suggesting an active engagement of the cell cytoskeleton.^79^

However, the weak interactions (i.e., van der Waals forces and electrostatic forces) securing the superficial bond between biomolecules and CPs make the physical absorption somewhat unreliable.^16^ For this reason, covalent bonding may be preferable, since it guarantees stronger protein–CP interactions.^16^ In most cases, for biomimetic adhesive molecules to be covalently attached to a CP film, several experimental strategies must be put in place to introduce active functional groups that would increase polymers reactivity and allow their biofunctionalization.^16,80−84^ For example, a chemical modification of PEDOT prior to polymerization might provide reactive carboxylic groups on the polymer surface to promote the attachment of laminin-derived peptides, facilitating cell adhesion.^81^

Another route to CPs functionalization for bio-interface applications is through the incorporation of bio-adhesive molecules in the polymer matrix.^63,85^ When polymers are synthesized by oxidation of the monomer, the concomitant incorporation of a negatively charged dopant is used to neutralize the positively charged polymer, stabilizing its backbone.^9,86^ Therefore, biomolecules bearing negative charges can act as biodopants when embedded into the polymer matrix. Following this approach, ECM-derived glycosaminoglycans such as chondroitin sulfate, hyaluronic acid, heparin and dextran sulfate, have been shown to influence C2C12 and myoblasts adhesion when embedded in PPy films.^87^ Additionally, each functionalization was shown to have a different impact on myoblast differentiation depending on the PPy thickness and surface morphology, highlighting the cell-specificity potential of these biomimetic approaches.

Furthermore, polymer/biomolecule blends can be used to improve the cell–chip electrical coupling when used as biomimetic coating on inorganic electrodes.^85,88^

Inspired by the native ECM, which also serves as a reservoir for growth factors, biofunctionalization strategies can also provide chemical cues other than that provided by bio-adhesive molecules, enriching the number of PM-mediated intracellular cascades and cell processes that can be effectively activated at the interface. For instance, Green et al. showed that the doping of a PEDOT film with *p*-toluenesulfonate (pTS), in combination with nerve growth factor (NGF), provides a softer biomimetic interface able to secure PC-12 cells adhesion, while activating specific transmembrane receptors which consequently induce cell differentiation and neurite outgrowth, confirming that the growth factor was still biologically active.^89^ Furthermore, as ECM-stored growth factors can be locally released and generate rapid and extremely localized signals,^90^ electric stimulation can be employed to induce the release of growth factors incorporated in CPs as dopants, controlling their spatiotemporal activity and presentation.^91,92^ Further details on functionalization strategies, cell types, and applications are presented in Table 2.

**Table 2:** CP-Based Chemical Functionalization Approaches

material	functionalization type	cell type	application	electrical stimulation	ref
PEDOT/xylorhamno-uronic glycan (XRU84)/fibrinogen/collagen/transferrin	physical absorption	human dermal fibroblasts	cell adhesion and spreading		(78)
PEDOT/PEG_10_-CDPGYIGSR (laminin-derived peptide)	covalent bond	rat pheochromocytoma (nerve) cells (PC-12)	cell adhesion and neurite extension		(81)
titanium coated with PPy/RGD	covalent bond	neonatal rat calvarial osteoblasts	cell adhesion		(83)
PPy/chondroitin sulfate (ECM molecule)/collagen-IV	biodopant–covalent bond	rat pheochromocytoma (nerve) cells (PC-12)	cell differentiation	yes	(82)
PPyCl/ ϕT59 (phage-derived peptide) /GRGDS	biodopant–covalent bond	rat pheochromocytoma (nerve) cells (PC-12)	cell adhesion		(80)
ITO coated with PPy/RGD	biodopant	human lung cancer cell A549	cell adhesion and proliferation		(88)
gold coated with PPy/CDPGYIGSRGold coated with PPy/SLPF (silk-like polymer)	blend	rat glial cells and human neuroblastoma cells	cell adhesion		(85)
gold coated with PPy/pTS/NT3 (neurotrophin-3)	blend	spiral ganglion neuron explant cultures	neurite outgrowth	yes	(91)
gold coated with PPy/pTS/BDNF	blend	spiral ganglion neuron explant cultures	neurite outgrowth	yes	(92)

Changing the redox state of CPs upon electrochemical doping/dedoping, can also be employed to induce conformational changes in ECM proteins exposed at the polymer surface. For instance, the application of an electrical potential can induce a conformational change of fibronectin when adsorbed on a PEDOT:tosylate film. The voltage-dependent switch of the polymer to its oxidized state induced the shift of fibronectin from a cell-adhesive conformation to a cell-repellent one no longer accessible for integrin binding. The presence of a functional fibronectin on the reduced electrode surface, securing integrin activation, promoted focal adhesion formation in MDCK cells while the oxidization of the polymer, preventing integrin binding, showed a dramatic reduction in the number of adhering cells.^93^

### 2.3 Functional Biomembranes

The PM, as the primary interface between the cell and its surrounding, mediates cell communication with its microenvironment and with neighboring cells.^4,94^ In this section we will introduce how cell–substrate interactions can be improved with biomimetic strategies based on supported lipid bilayers (SLBs).

Furthermore, we will discuss how different techniques to form artificial membranes can be used to functionalize and disguise CP-based platform, simulating cell–cell interactions at the interface.

#### 2.3.1 Recapitulating PM Composition and Fluidity: General Considerations

Crucial for the cell communication with its surroundings, the peculiar structure of the PM is constituted by an asymmetric bilayer where the inner and the outer leaflets are made of different lipid molecules,^95,96^ while sterols and proteins are distributed within the membrane according to their hydrophilic/hydrophobic domains.^97^ More in detail, the membrane is subcompartmentalized in microdomains, known as lipid rafts: these highly ordered lipid and lipid-anchored proteins can be segregated laterally in the PM, allowing precise control over the localization of signaling biomolecules involved in intracellular cascades propagation^97,98^ as well as focal adhesion complexes formation.^99−101^ Lipid rafts also support the formation of cellular protrusions, further highlighting the central role of the PM in regulating cell adhesion and spreading.^102^

As the cell cytoskeleton responds and reshapes according to the fluidity of the substrates,^103^ biomimetic engineering approaches have been introduced to emulate the PM structure within in vitro bio-electronic platforms, mirroring cell–cell interaction at the cell–chip interface.^104^ Among several models implemented to resemble the complex nature of biological systems, SLBs allow one to recapitulate the lipid rafts structure and dynamicity of the PM.^105^ In particular, the possibility to tune the SLBs composition to directly influence their properties, such as the bilayer fluidity, has emerged as a crucial point in interfacing the cell PM and inducing the activation of attachment sites. In fact, rigid SLBs, by providing more anchoring points for focal adhesion proteins recruitment, were shown for instance to successfully promote neural stem cell adhesion.^106^ The integrin-mediated reorganization of the cytoskeleton was also confirmed by cells’ stretched and elongated morphology and mediated neural stem cell differentiation.^106^

In order to increase the SLB biomimetic potential, additional functionalization with adhesive biomolecules have been implemented. In this case, the artificial membrane requires the presence of lipids with reactive groups exposed on the outer leaflet to covalently bind ECM proteins.^107,108^ Furthermore, the fluidity of the SLB allows surface ligands to diffuse freely within the bilayer^109−111^ contributing to the proper assembly of membrane protein clusters which naturally occur in cells in response to traction forces exerted by the PM.^112−114^

#### 2.3.2 Formation of Supported Lipid Bilayers

**Figure 4:**
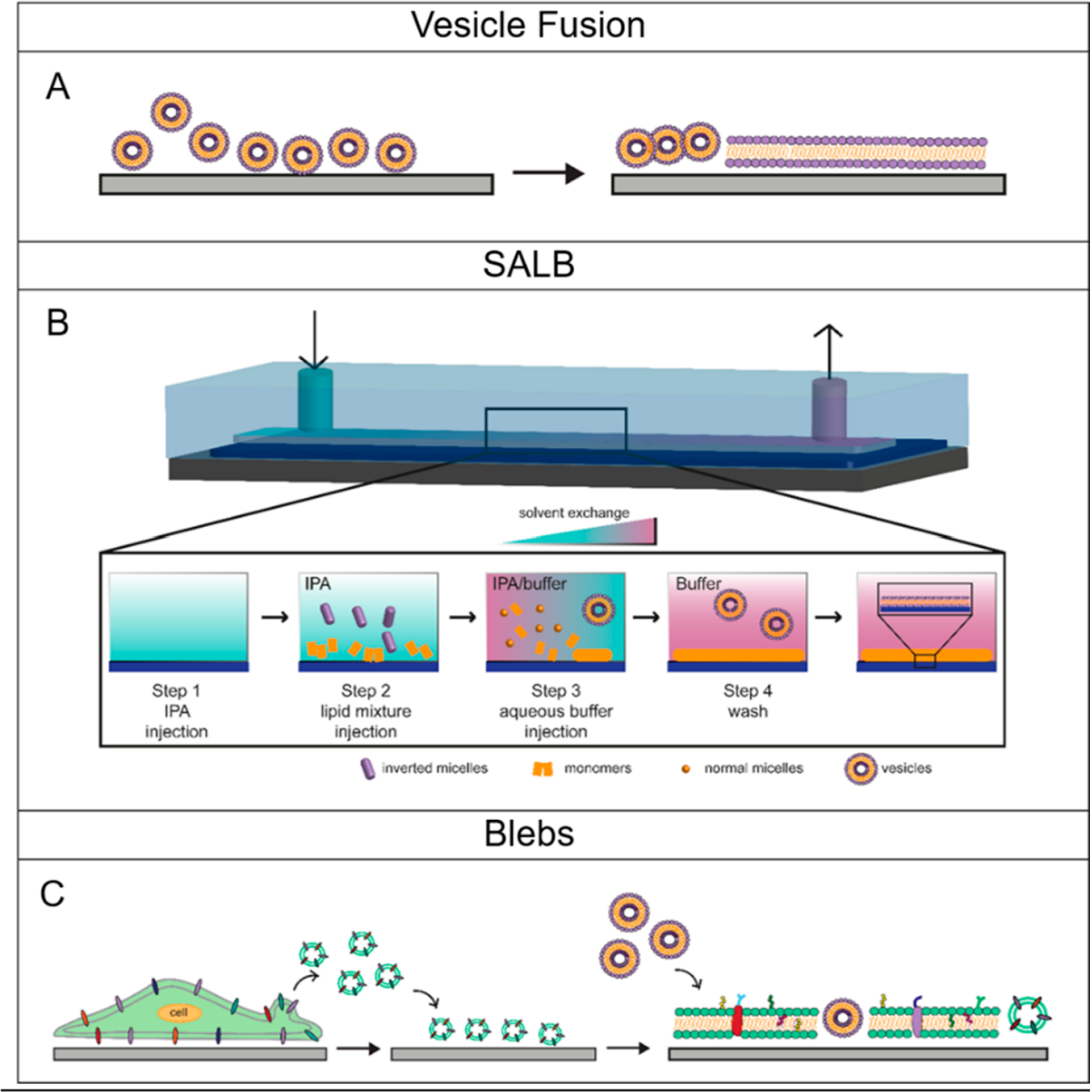
SLBs formation strategies. Schematics depicting the possible approaches to obtain artificial membranes: (A) vesicle fusion, with vesicles spontaneously fusing on solid supports; (B) solvent-assisted lipid bilayer, depicting vesicles rupture induced by solvent exchange within the microfluidic channel; and (C) blebbing, illustrating blebs collection from cell plasma membrane and consequent bilayer formation.

The biomimetic properties of SLBs with the possibility of preserving the PM biological structure and proteins native conformation gained particular attention in bioelectronics in order to characterize and monitor PM properties, to investigate the role of synthetic compounds on membrane functions^115−118^ and ultimately interface cells. In fact, when formed on conductive materials, these artificial membranes provide an insulating layer that might reduce leakage currents, thus improving electrical measurements.^119,120^ Vesicle fusion (VF) is the most widely employed technique to form SLBs. It is based on a self-assembly process where vesicles, through van der Waals and electrostatic forces, absorb on a solid support.^121,122^ Here, once reaching a critical surface coverage, vesicles spontaneously rupture and fuse to form the bilayer as shown in Figure 4A. The nature of this process makes it highly dependent on the material surface properties, and for this reason, it can only be applied on a narrow range of substrates as silica-based materials.^123^ Therefore, substrates suffering of poor wettability, i.e., gold, necessitate chemical modifications to provide adequate surface tension.^124^

Recently, a new strategy, namely, solvent-assisted lipid bilayer (SALB) method, has been proposed to support the formation of bilayers on diverse materials (i.e., gold, aluminum, oxide, silicon dioxide, graphene, and CPs), being not strictly limited by the material surface and lipids composition.^125−128^ In the SALB approach, the lipid vesicles adsorption and planar assembling on the substrate occurs inside a microfluidic channel, as shown in Figure 4B. Here, the solvent mixture of isopropanol and water, in which the lipids are suspended, is slowly replaced with an aqueous buffer, minimizing the effect of the surface tension at the lipid–substrate interface.^125^ Furthermore, both VF and SALB are suitable techniques for the functionalization of the bilayer with ECM proteins and biomolecules either embedded within the double layer^129−131^ or covalently tethered to the lipid headgroups.^125^ Zobel et al.,^130^ for instance, incorporated *N*-cadherin and ephrinA5 proteins, well-known synaptic modulators,^132^ into supported lipid bilayers to resemble the composition of a synaptic niche. Here, primary neuronal cells showed a significant increase in neurite activation and extension when cultured on the *N*-cadherin-doped artificial membranes, highlighting the validity of this approach in the precise characterization of protein-mediated cell function.^130^

While embedding single proteins within a SLB is a powerful tool to investigate specific interactions at the interface, this approach is far from replicating the complex structure of actual cell membranes.

In this scenario, recently SLBs have been engineered with blebs obtained from different cell types by chemical treatment,^133^ as shown in Figure 4C. The blebbing mechanism is regulated by the mechanical properties of the actin cytoskeleton, whose local contraction leads to the formation, growth, and detachment of these quasi-hemispherical protrusions.^134^ So far SLBs constituted from cell-derived blebs, have been mainly exploited for biosensing to investigate proteins activity in their native environment.^127,135^ In addition to this, blebs-based SLBs enriched with biochemical cues appear also as emerging platforms in bio-electronics able to engage a tight contact with living systems as recently proven for cardiomyocytes, where the presence of the biomimetic membrane preserves cell–cell interactions and cardiac cell contraction ultimately promoting the formation of a beating tissue.^136^

#### 2.3.3 SLBs on Conductive Polymers

The translocation of proteins and their conformational change within the cell PM is essential for the proper assembly of adaptor and signaling molecules.^137^ However, in artificial membranes, such mechanism is hindered by the frictional coupling between protein protruding domains and rigid surfaces which leads to protein denaturation and loss of activity.^137^

For this reason, the development of tethered bilayers and polymer-supported SLBs have been introduced: here, either the presence of a spacer between the inner leaflet and the solid support, or the cushioning effect provided by the polymeric layer, are crucial to preserve proteins’ native conformation.^138−142^

In this scenario conductive polymers, due to their intrinsic soft properties,^143^ are ideal candidates to reduce the frictional coupling with embedded proteins, while their conductivity also allows for the electrical characterization of the obtained SLBs as well as monitoring ligand–proteins binding.^144^

However, the formation of SLBs with VF has so far been hampered by the hydrophobic nature of CPs and by the presence of negatively charged dopants,^145^ limiting the number of possible membrane compositions that can be homogeneously formed on such substrates. In this scenario, the development of the SALB technique enabled the formation of various artificial membranes types (ranging from bacteria to mammalian cells) on soft CPs, also preserving the native conformation and functionality of integrated proteins.^125^

Following this approach, recently CPs have been engineered with different models of cell membranes as bacterial and mammalian membranes to investigate the effects of pore forming toxins and antibiotic compounds,^144^ but also to monitor the activity of transmembrane proteins and ion channels.^127,146,147^ Indeed, the mixed ionic electronic conduction of CPs provides a direct monitoring of the proteins activity, either in their native conformation or upon drug treatments.^127,147^ Therefore, such CP–biomembrane platforms appear as highly promising candidates for being interfaced with living cells mimicking stiffness, fluidity, and composition of biological systems.

### 2.4 Exploiting Intrinsic Electrical Properties of CPs to Characterize Planar Cell–Material Interfaces

In this section we will discuss how intrinsic properties of CPs may be used to enhance sensing capabilities of both passive electrodes and three-terminal devices in order to characterize cell processes at the interface through electrical and optical measurements.

#### 2.4.1 Impedance

Processes that regulate the interactions at the interface between cells and planar electrodes can be characterized by monitoring certain electrical parameters over time. One of these is the electrochemical impedance, measured through electrochemical impedance spectroscopy (EIS), a technique used to assess the frequency response and surface-dependent properties of a system upon the application of an alternate voltage.^148^ EIS can be exploited both for inorganic^149^ and organic electroactive materials as CPs^88,150^ as, in general, conductors allow for the passage of charges, through resistive paths, after the application of an alternate/fixed voltage. Biological tissues, on the other hand, consist of insulating layers, such as the PM, that behave like leaky capacitors, i.e., devices that accumulate charges on the surface, still allowing for a leakage current to cross the layer itself.^151^

**Figure 5:**
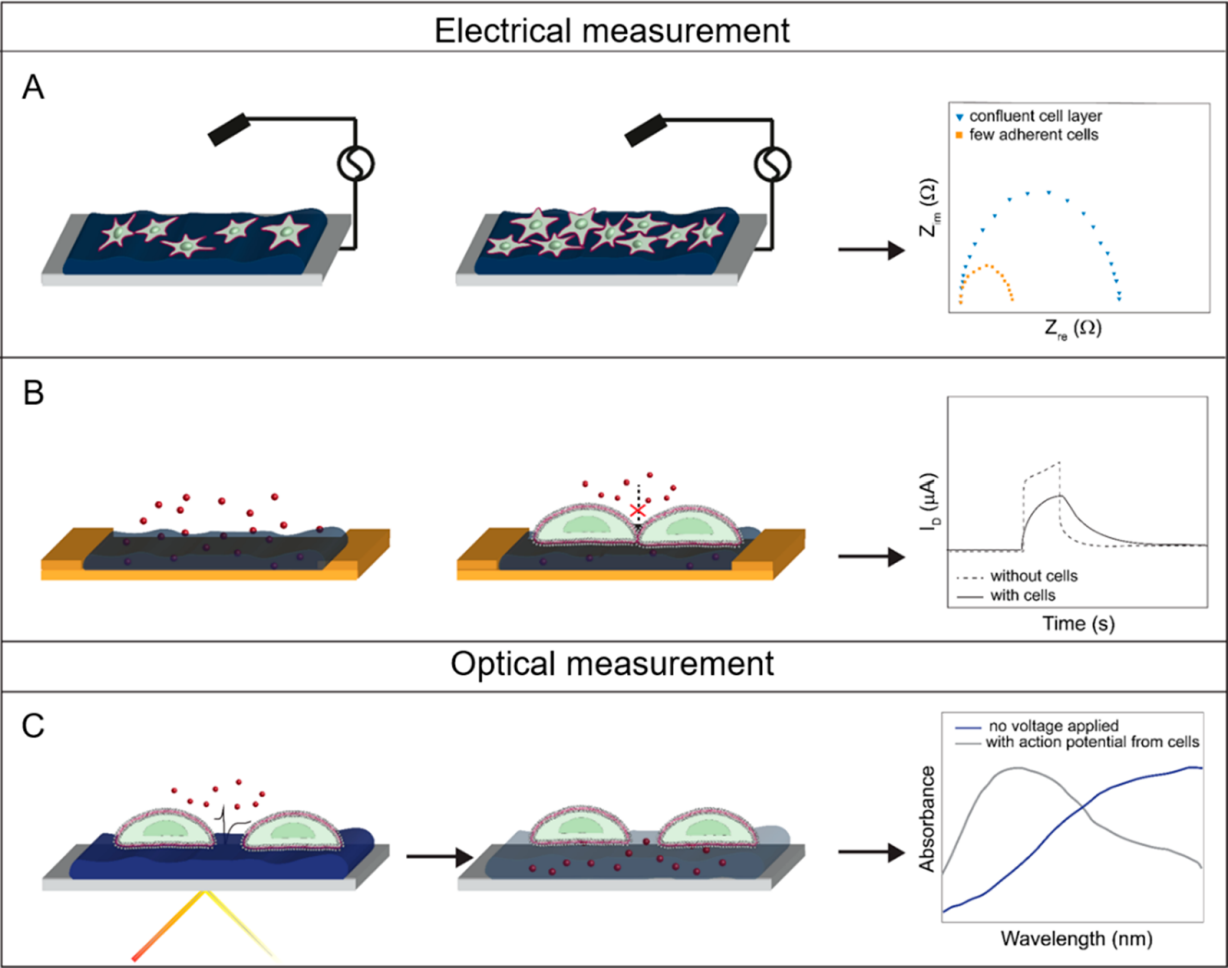
CP-based approaches for the cell–material interface characterization on 2D systems. Schematics showing (A) electrochemical impedance spectroscopy with a Nyquist plot, (B) electrical modulation of an OECT response mediated by cell monolayer formation, and (C) shift in the absorbance peak of PEDOT:PSS upon the application of an external voltage.

Importantly, the high sensitivity of this technique to several electrochemical mechanisms (e.g., diffusion, electron transfer rate, or absorption mechanisms) paves the way for a plethora of bioanalytical applications,^148^ including the real-time monitoring of a cell monolayer formation through the estimation of equivalent electrical parameters. In fact, the electrical behavior of phospholipid membranes can be described by two lumped parameters: a capacitor and a charge transfer resistance, which model the accumulation and the passage of charges, respectively.^148^ In particular, cell adhesion and proliferation on the substrate can influence the number of ions that freely reach the surface, modulating the resistive component of EIS.^152^ On the other hand, cell–cell interactions, such as tight junctions’ formation, can affect the equivalent capacitance.^153^ Similarly, the formation of intact homogeneous artificial membranes on CPs can be confirmed by monitoring variations in impedance components,^127,144,146,147,154^ as shown in Figure 5A.

#### 2.4.2 Carriers Mobility

CPs, being sensitive to ionic charges, emerged as leading materials for OECTs, three-terminal devices in which a source and a drain electrode are connected by an organic semiconductor channel. Their electrical operation depends on the injection of ions from an electrolyte into the bulk of the organic channel which modulates its conductivity through electrochemical doping.^155^ Thanks to this carriers’ mobility, these CP-based OECTs found extensive application as biosensors to continuously monitor biological processes at the interface. For instance, the OECT in situ signal amplification, improving signal-to-noise ratio, enables fast and precise recordings from electrogenic cells,^156−159^ or the detection of biological molecules, including neurotransmitters.^160^ Furthermore, different processes, such as the formation and disruption of tight junctions in cell-barrier tissues, can be monitored through the modulation of the ions propagation rate which determines an increase in the OECT response time (τ), evaluated from the real-time monitoring of the channel current upon the application of square voltage pulses at the gate electrode, as shown in Figure 5B. Consequently, the disruption of tight junction proteins, through the ethanol-induced poration within the cell layer, showed a complete recovery of the initial τ.^161^

Similarly, the formation of a cell layer on the OECT channel can be evaluated through the measurement of the transconductance, a parameter describing the voltage-to-current transduction factor of the transistor,^162^ or by monitoring the shift in the frequency response of the device, allowing for single-cell level detection.^153^

In addition, OECTs can also be employed to study more complex mechanisms at the interface which involve membrane proteins and their activation upon drug treatment.^127,146^ The investigation of transmembrane proteins activity gained particular attention in bio-electronics and cell interface biosensing. The ability of such proteins to transport ions and small molecules across the PM establishes a direct communication between external electronic devices and intracellular compartments, making them ideal targets for drug screening applications.^163,164^

Recently, the possibility to integrate SLBs with OECTs has significantly contributed to the characterization of the behavior of membranes and embedded proteins. Such biomimetic transducers have been employed to study the activity of the TREK-1 ion channel (i.e., K^+^ channel responsible for controlling cell excitability) embedded in a SLB:^146^ in the presence of a K^+^ blocker, the ion channels have a closed conformation that inhibits the passage of ions through the artificial bilayer. The different rate of crossing ions results in a different modulation of the carrier mobility in the CP channel of the transistor.^155^ This effect is confirmed by the increased τ of the OECT. On the other hand, by opening the channels with a TREK-1 activator, the ions can freely diffuse through the SLB, thus modulating the conductance of the OECT and restoring the initial response time of the device. Such biomembrane-based sensors open the possibility to explore several types of transmembrane proteins (i.e., ligand-gated ion channels^127^) or mechanosensitive channel protein^165^ and investigate their behavior in response to topographical, mechanical, and chemical cues.

#### 2.4.3 Electrochromism

**Figure 6:**
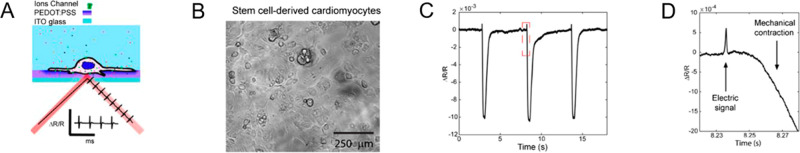
CP-based electrochromic platform to record electrical activity of excitable cells. (A) Schematic of the optical setup. (B) Bright-field image of cardiomyocytes cultured on a PEDOT:PSS film. (C) Fractional reflectivity change signal obtained by optical recordings of cardiomyocytes. (D) Zoom in of panel C showing the electrical signal as a small spike occurring 15 ms before the mechanical contraction of cardiomyocytes. Adapted with permission from ref (166). Copyright 2020 National Academy of Sciences.

Cell processes regulated by ionic exchanges, such as action potentials, can be further investigated exploiting the electrochromic properties of CPs,^166^ as schematically shown in Figure 5C. Indeed, the delocalization of electrons along the polymer backbone and the dopant counterions account for the formation of colored compounds as in the case of PEDOT:PSS, easily recognizable from its dark blue color.^167^ The injection of ions upon dedoping, however, opposes the electronic conjugation between the polymer and the doping agent, causing a color variation in the polymer film.^167^ Recently, Alfonso et al. implemented an electrochromic platform which exploits the dedoped transmissive state of PEDOT:PSS to optically record action potentials.^166^ Here, the cells provide a local bias to the polymer able to induce dedoping and resulting in a variation in the absorption of the film, allowing optical visualization of the action potential generation without the addition of external electrodes or fluorophores, as shown in Figure 6.

## 3 Engineering the Electrical Bio-interface with Pseudo-3D Nano- and Microstructures

### 3.1 Biomechanical Processes Regulating the Interaction of Cells with Pseudo-3D Materials

Inspired by the shapes and geometries found in the ECM with its fibrils, pits, and posts, engineered micro- and nanoelectrodes, such as nanoholes, grooves, and pillars, have recently emerged as promising candidates in designing biomimicry strategies for bio-interface optimization.^168−170^

**Figure 7:**
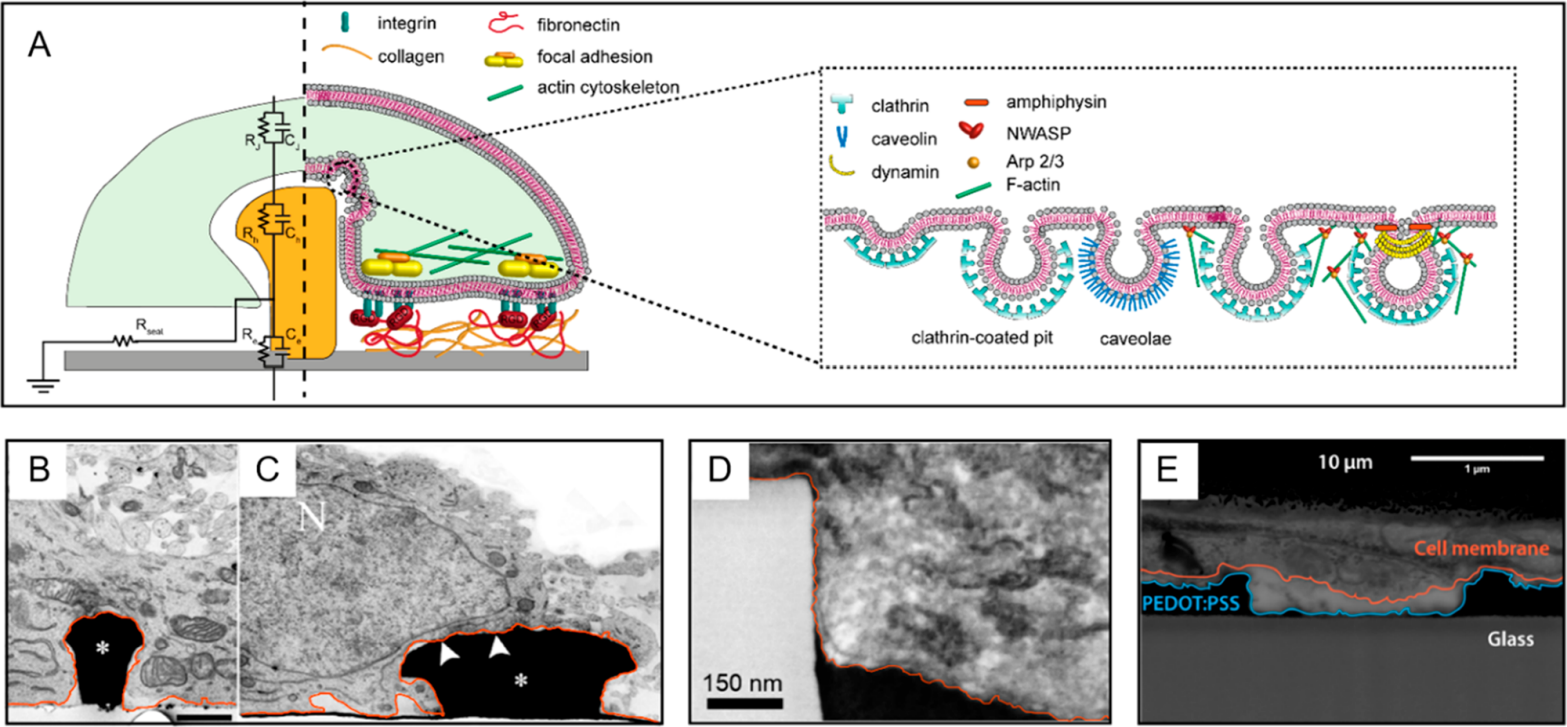
Cell–electrode interactions in pseudo-3D systems. (A) On the left, electrical equivalent circuit modeling the cell–electrode interface; on the right, schematic representation of the integrin-mediated cytoskeletal activation and focal adhesion proteins maturation describing the spatial relationship between a cell and a pseudo-3D electrode. The inset shows the topography-driven engagement of the cell endocytic machinery. Transmission electron micrograph showing the tight engulfment of a mushroom-shaped electrode by a neurite (B) and a cell body (C). (D) FIB-SEM cross-section showing the tight interface with a PEDOT-coated quartz nanopillar. (E) FIB-SEM cross-section showing the PM and local ruffling on a grooved PEDOT:PSS substrate. Orange line and arrows indicate the PM profile. Blue line indicates the PEDOT:PSS substrate profile. Reprinted and adapted with permission from the following: Ref (175). Copyright 2015 The Authors under Creative Commons Attribution 4.0 International License, published by Springer Nature. Ref (29). Copyright 2017 American Chemical Society. Ref (176). Copyright 2017 American Chemical Society.

The ability of the cell to constantly and dynamically rearrange its PM and cytoskeletal architecture to wrap around vertically aligned structures (e.g., dendritic spines and filipodia) can be exploited to promote engulfment-like events at the cell–material interface, allowing the cell to wrap around purposely designed pseudo-3D electrodes,^171−174^ as schematically shown in Figure 7A.

In fact, the fluid nature of the PM serves as an ideal environment to trigger a phagocytic-like event and accommodate a protruding electrode, as shown in Figure 7B–E. The curvature-induced PM sorting^177−180^ allows one to tune the localization of transmembrane proteins, including integrins,^181,182^ and the formation of specific domains that might locally enforce the activation of signaling pathways involved in the activation of the cytoskeletal machinery,^183^ as shown in Figure 7A. One of the first observations of the contribution of the cell cytoskeleton to the cell–chip coupling in pseudo-3D systems was proposed by Hai et al. using mushroom-shaped electrodes designed to recapitulate dendritic spines’ shapes and dimensions. Here, the stabilization of the cell–electrode interface is achieved by the topography-induced formation of organized actin rings around the stalk of the engulfed microstructure.^168,184−186^ The ability of protruding electrodes to induce the shape rearrangement necessary for the cell to accommodate the pseudo-3D structures was further confirmed by recent observations on the topography-driven recruitment of curvature sensitive proteins (e.g., Bin/Amphiphysin/Rvs (BAR) proteins) at the cell–material interface,^177,187^ as shown in Figure 7A. Among these, F-actin typically accumulates at nanopillars tips,^188^ where high positive curvature is strongly induced by the pseudo-3D geometry.^189^ Here, further studies highlighted that the curvature-dependent arrangement of actin is associated to the expression of Nadrin-2, a regulator of actin polymerization, and FBP17, a mediator of actin nucleation, that also co-assembles at the tip of vertically aligned nanostructures.^190^ In particular, the recruitment of curvature-sensing FBP17 causes the activation of downstream signaling components including N-WASP, cortactin, and the Arp2/3 complex and initiates the nucleation of branched F-actin.^189^ This active actin network influences how the cell membrane rearranges to accommodate pseudo-3D topographical cues, with a more stable interface being formed when the center of the cell is engaged by vertically aligned structures.^191^

PM reshaping is also supported by the curvature-induced PM sorting of the scaffolding structures involved in clathrin- and caveolae-mediated endocytosis,^192^ as shown in Figure 7A, which contribute to the relocation of membrane patches through the rapid uptake of membrane and its re-insertion at sites of membrane extension or deformations.^193,194^ Notably, high-aspect-ratio nanostructures have been shown to activate the curvature sensitive BAR protein Amphiphysin 1, a regulator of endocytosis, at the cell–material interface.^190^ In addition, endocytic proteins (e.g., clathrin, caveolin-1, and dynamin) have been found to accumulate on nanoscale-curved membranes^188,195−197^ while clathrin-coated pits and caveolae formation was revealed at the cell–material interface by electron microscopy techniques,^171,195−197^ highlighting how the curvature-induced activation of the cell endocytic machinery might be essential in mediating the phagocytic-like event of pseudo-3D electrodes.

In this scenario, further chemical functionalization with engulfment promoting peptides, such as the fibronectin-derived RGD peptide or the laminin-derived PA22-2, can be used to promote engulfment-like events and induce stronger connection between cells and pseudo-3D electrodes.^173,174,198^ Furthermore, biofunctionalization approaches can be used to guide cellular distribution on specific restricted areas of a substrate: for instance, bioadhesive poly dl-ornithine (PDLO) was used to specifically guide cell attachment on vertical nanopillars where the interactions between the topographical cues provided by these pseudo-3D structures enhanced synapses formation and maturation.^199^ Similarly, a patterned thiol-based self-assembled monolayer, exploiting the attraction between the positively charged amino groups of the coating and the negatively charged PM, can be used to specifically distribute cells on the gold surface of mushroom-shaped microspines.^200^

### 3.2 Cell–Surface Electrode Modeling in Pseudo-3D Systems

The biomechanical reshaping of the PM at the interface between cells and pseudo-3D substrates was shown to drastically reduce the cleft^17,29,201^ going from 40 to 100 nm, observed in planar substrates, to 5–20 nm.^29^ In addition, a volumetric and more complex interaction between the PM and the pseudo-3D electrode must be considered, as shown in Figure 7A. The cell membrane, in fact, may or may not entirely engulf a pseudo-3D electrode, forming hourglass-like or tent-like profiles, depending on the geometry of the electrode itself.^191,202^

**Figure 8:**
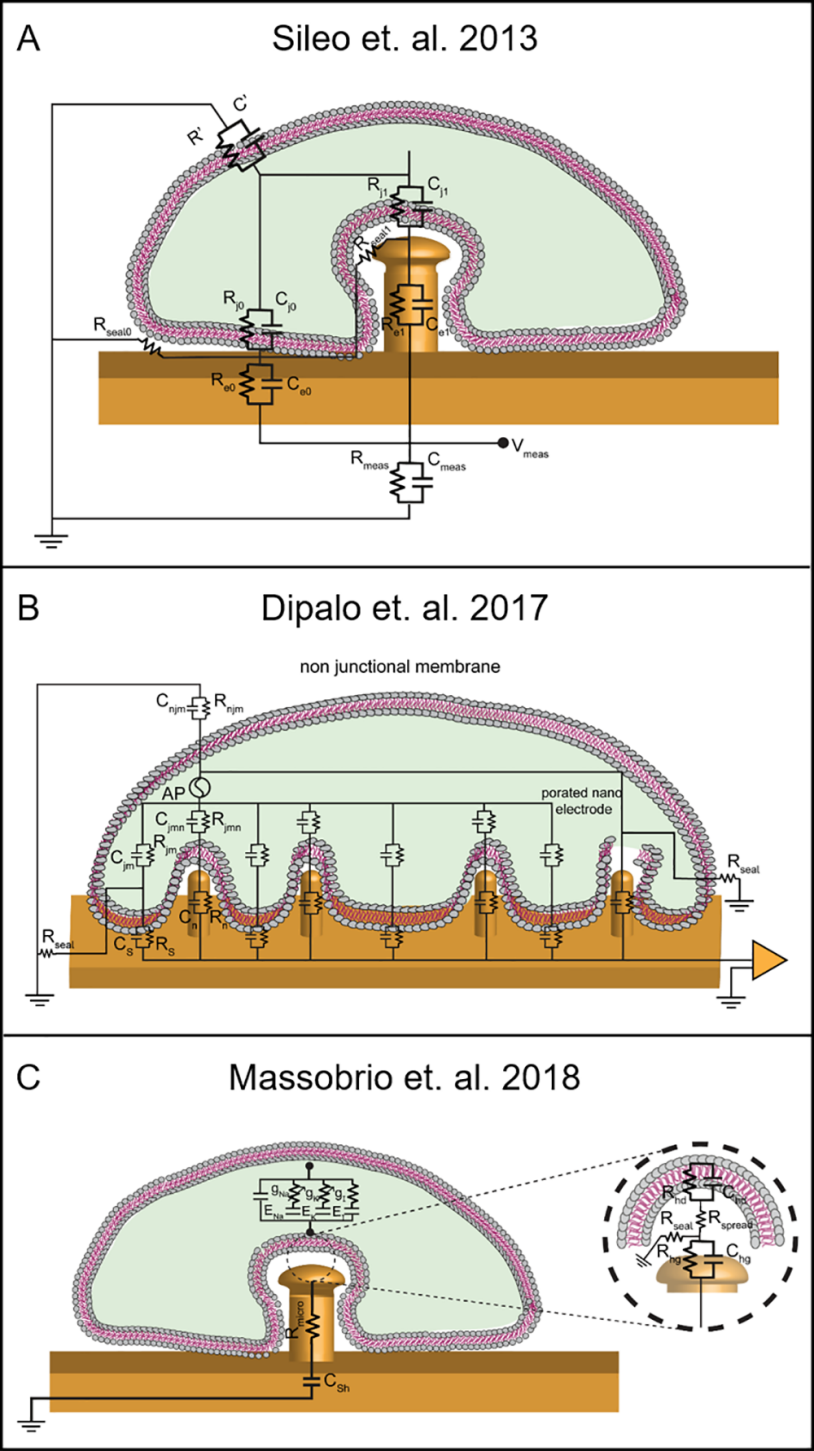
Electrical modeling in pseudo-3D systems. Schematic representation of the electrical equivalent circuit modeling proposed by (A) Sileo et al. in which the coupling at planar and pseudo-3D domains of the electrode is accounted for through different sealing resistances, (B) Dipalo et al. showing an array of vertically aligned structures modulating the coupling for each part of the electrode, and (C) Massobrio et al. in which the cell–chip coupling is characterized by the modeling of the formation of the electrical double layer (EDL) on electrode surface (*R*_hg_ and *C*_hg_), along with a single sealing resistance computed by considering different contributions due to the geometry of the electrode itself. Reprinted or adapted with permission from the following: Ref (203). Copyright . Ref (204). Copyright 2017 American Chemical Society. Ref (205). Copyright 2018 IEEE.

In the light of this, a new bioelectric model was proposed by Sileo et al. to elucidate the cell-electrode arrangement arising from the pseudo-3D architecture,^203^ as shown in Figure 8A. Here, in the case of a single pseudo-3D protrusion, the interface is described by the addition of a parallel *R*–*C* circuit to the previously proposed model for planar electrodes,^40^ to address the added complexity of the interfacial interactions between cells and a pseudo-3D electrode. Moreover, two sealing resistances (*R*_seal1_ and *R*_seal2p_) were appraised considering the clefts’ width resulting from the coupling mechanisms simultaneously taking place at the planar and pseudo-3D interfaces.^203^ Dipalo et al. proposed a similar model (Figure 8B), in which an array of pseudo-3D microelectrodes are coupled with a single cell, and the overall coupling strength might be modulated by a collective equivalent sealing resistance (*R*_seal_).^204^ A different modeling approach was proposed by Massobrio et al., where a single mushroom-shaped microelectrode was coupled to the PM and the equivalent electrical components (*R*_seal_, *R*_e_, *C*_e_, *R*_h_, and *C*_h_) were calculated considering diverse types of contact occurring at different electrode regions, as shown in Figure 8C. Thus, the model included distinct sealing resistance contributions depending on the geometrical parameters overruling the cleft at the stalk and cap of the mushroom-shaped electrode.^205^ Recently, efforts were made to transit from a parametrical formulation of the coupling to a model based on mathematical functions that account for dynamic processes, i.e., membrane reshaping and wrapping, at the pseudo-3D–cell interface^53^ or nanometer-scale deformations observed during action potentials.^206^ Here, the possibility to describe the continuous variation of the coupling conditions allows for the analysis and the prediction of the electrode interaction with cells, enabling the optimization of vertically aligned electrodes design prior to experimental validation.

### 3.3 Pseudo-3D “Passive” Bio-interfaces for Electrophysiology

The potential of pseudo-3D inorganic electrodes to induce PM local reshaping has found several applications in the electrophysiology field and inspired CP-based pseudo-3D platforms. In fact, the top rearrangement at the cell–material interface and the consequent reduction of the cleft guaranteed by biomimetic mushroom-shaped electrodes, allowed Hai et al. to record “intracellular-like” signals from *Aplysia* buccal neurons, enabling the acquisition of attenuated action potentials and subthreshold synaptic potentials like those achieved with the patch clamp technique.^17,168,201,207,208^ Furthermore, the biomimetic potential of mushroom-shaped electrodes has also been employed to guide and simultaneously record from HL-1 cells^209^ and for optical detection of action potentials.^210^

**Figure 9:**
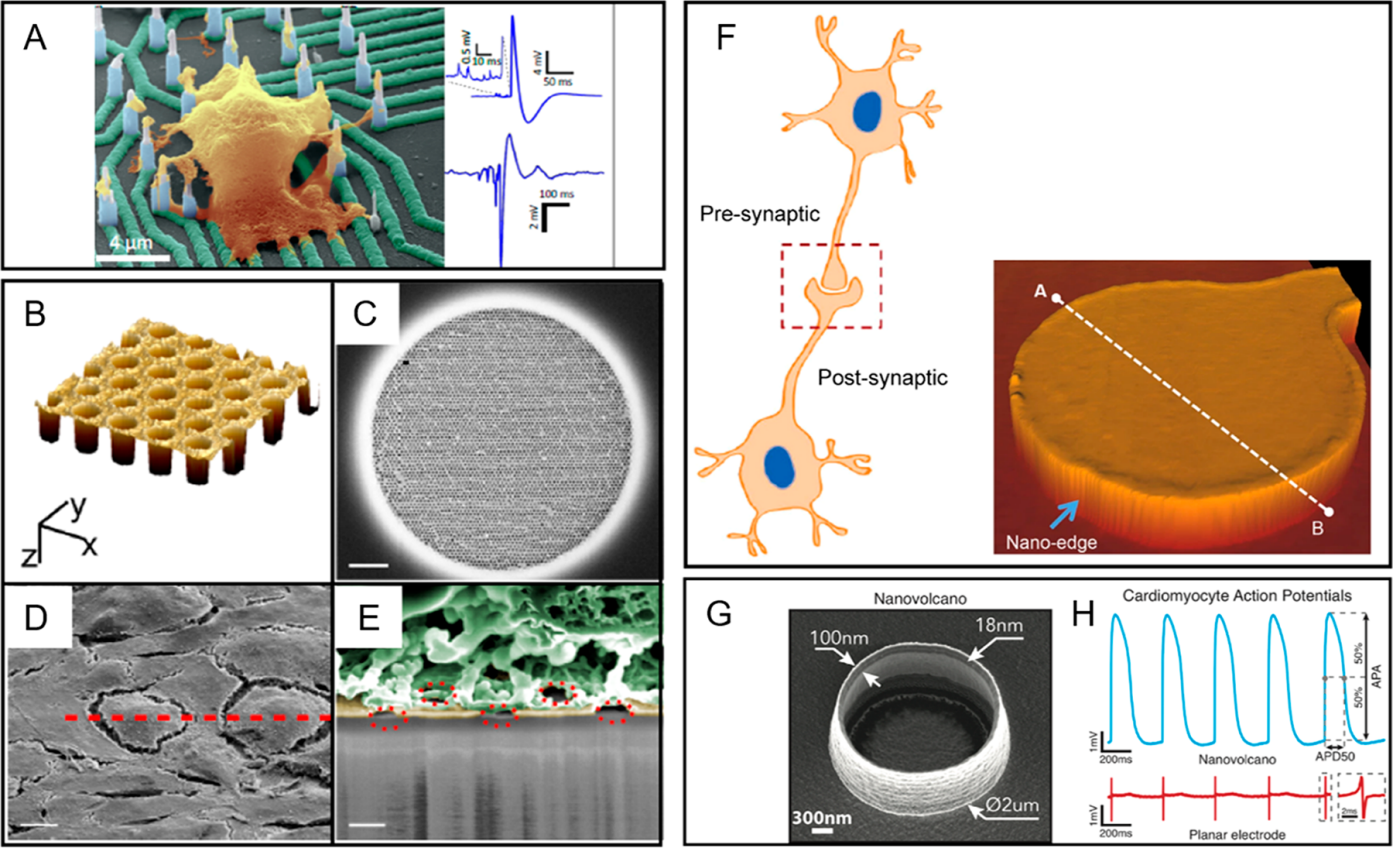
Pseudo-3D bio-interfaces for electrophysiology. (A) Colorized scanning electron micrograph showing a hiPSC-derived cortical neuron growing on a nanowire array (scale bar: 4 μm). (B) Atomic force microscopy image of a holey gold microelectrode. (C) Scanning electron micrograph of a holey array (scale bar: 4 μm). (D) Scanning electron micrograph of HL-1 cells on holey gold (scale bar: 8 μm). (E) Colorized scanning electron micrograph of a FIB cross-section (scale bar: 200 nm). The rough areas are colored in yellow and the cell is colored in green to provide better contrast of the cell–holey gold interface. (F) Nanoedged microelectrode recapitulating the morphology of a synaptic cleft. The blue arrow delimits the nanoedged perimeter of the microelectrode. (G) Scanning electron micrograph of a nanovolcano. (H) Intracellular recording of action potentials (upper trace) and simultaneous electrograms from a planar electrode (lower trace). Reprinted and adapted with permission from the following: ref (214). Copyright 2019 American Chemical Society. Ref (170). Copyright 2019 American Chemical Society. Ref (212). Copyright 2017 American Chemical Society. Ref (169). Copyright 2016 The Authors under Creative Commons Attribution 4.0 International license, published by Springer Nature.

If engineering planar devices with pseudo-3D electrodes has been important in enabling in-cell recordings, when the same approach was applied to record signals from mammalian cells, the engulfment of the mushroom-shaped electrodes produced a loose seal-like configuration (juxtacellular configuration), resulting in a reduced amplitude and shorter duration of the recorded signals.^211^ These results suggest that the stark difference in size between invertebrate and mammalian neurons can indeed influence engulfment and has to be accounted for.^53^ In this scenario, vertical nanowire MEAs, with their smaller size, can be used to continuously record from single cells or to achieve subthreshold sensitivity by increasing the nanostructure density,^206^ as shown in Figure 9A.

Following a similar biomimetic design of electrodes, Wijdenes et al. developed a nanoedged microelectrode mimicking the peculiar morphology of the synaptic cleft where the pre- and post-synaptic terminals are juxtaposed, and the presynaptic terminal is partially encapsulated by the postsynaptic button, as shown in Figure 9F. These simple nanoscale structural modification of the electrode, increasing the active surface in contact with the cell, resulted in a significantly improved electrical seal between neurons and the device itself, also enabling long-term recordings at single-neuron resolution.^169,212^

Similar results in the further decrease of the cleft and in the increase of the area of the electrode interfacing with the cell were achieved with nanotubes with a hollow center: the protrusion of the cell PM inside the nanostructures’ pits, confining ion channels flux in the small membrane patch within the electrode and preventing ions’ leakage through cleft, resulted in improved quality of recordings.^213^ Lastly, similar hollow-structured pseudo-3D electrodes, such as nanoholes (Figure 9B,C) and nanovolcanoes (Figure 9G), were exploited to encourage the spontaneous suction of the PM, further tightening the cell–chip sealing (Figure 9D,E) and improving recordings quality (Figure 9H).^170,212,214,214,215^

### 3.4 Active Engagement and Modulation of the Pseudo-3D Bio-interface

**Figure 10:**
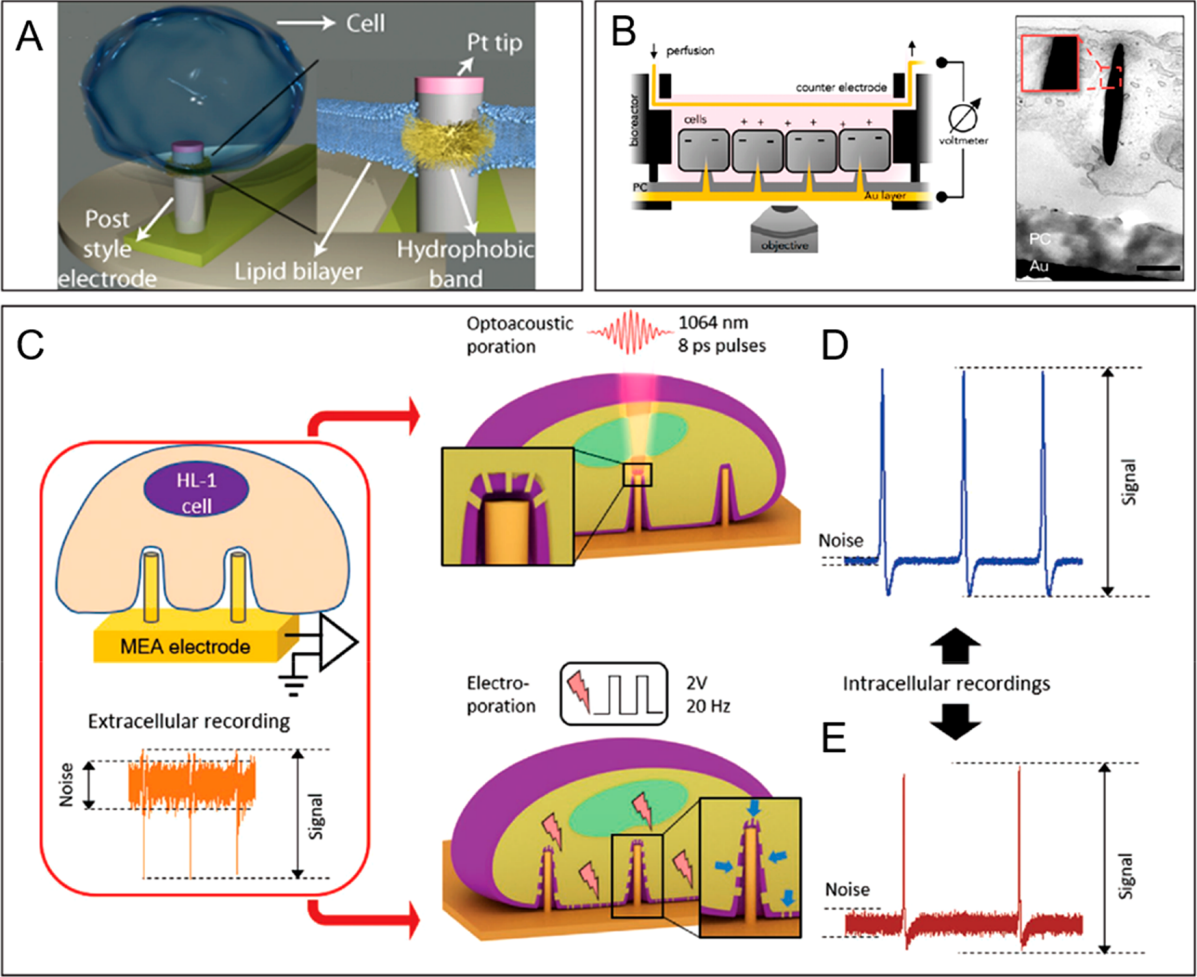
Pseudo-3D active bio-interfaces. (A) Schematic representation of the “stealth probe” fused with the cell membrane and a magnified view of the bio-interface. (B) On the left, schematic representation of cell monolayers interfaced with gold nanoelectrodes; on the right, transmission electron microscopy image revealing the close contact between the cell PM and the electrodes. The inset depicts a higher magnification of the electrode–cell interface (scale bar: 200 nm). (C) Schematic representation of the nanostructures used to deliver electroporation and optoacoustic poration. The optoacoustic stimulation induces the formation of pores of the size of the laser focus volume, whereas electroporation affects the PM along the whole contact region. Intracellular recordings obtained following (D) optoacoustic poration and (E) electroporation. Reprinted and adapted with permission from the following: Ref (224). Copyright 2010 American Institute of Physics. Ref (228). Copyright 2019 American Chemical Society. Ref (237). Copyright 2019 Wiley.

The tight apposition of cells to pseudo-3D structures and the topography-induced high local PM deformation can lead to local rupture and effective poration^216^ to gain intracellular access for sensing and drug delivery applications.^217−220^ Moreover, in order to promote PM penetration, vertically aligned nanostructures may be functionalized with diverse biomolecules such as cell penetrating peptides (e.g., the trans-activating transcriptional activator (TAT) from human immunodeficiency virus 1)^221^ or positively charged compounds that facilitate the interaction with the negatively charged PM domains.^222^ But spontaneous PM permeabilization can also be encouraged by functional coating with SLBs: sub-10 nm nanowire–nanotubes probes with a phospholipid coating were shown to penetrate and retract from HL-1 cells in a minimally invasive manner, enabling continuous intracellular and/or extracellular recordings. Importantly, these ultrasmall probes, which approach the size of a single ion channel, were able to record sub-millisecond physiological signals.^223^ The spontaneous insertion into the hydrophobic membrane core of interfacing cells can be achieved by membrane-inspired nanoscale electrodes, consisting of two hydrophilic posts separated by a hydrophobic band formed on a gold layer. This configuration, closely resembling the architectural organization of a lipid bilayer with its hydrophilic heads separated by a hydrophobic layer of closely packed lipid tails, contributed to the insertion of the electrode in the cell PM and in the formation of a tight interface, with spontaneous gigaohm seals formation,^224^ as shown in Figure 10A. However, there is still some debate as to if PM penetration spontaneously occurs,^222,225^ with studies suggesting that, in arrays containing hundreds of vertically aligned nanostructures, only a fraction of them actually penetrates an adherent cell.^222,226,227^ Furthermore, despite the recruitment of focal adhesion complexes and cytoskeleton rearrangement stabilizing pseudo-3D electrodes PM penetration,^228^ as in the case of gold nanoelectrodes shown in Figure 10B, the activation of PM repair mechanisms often leads to the exclusion of these structures, resulting in an unstable and transient penetration in most cases.^225,229,230^

For this reason, several techniques have been developed to secure a robust and durable intracellular access. Electroporation, for instance, is a technique that causes the formation of localized nanoscale pores at the cell–material interface through the application of an electrical field, as shown in Figure 10C, enabling PM penetration of pseudo-3D nanoelectrodes and intracellular electrophysiological recordings.^231−235^ The application of a small voltage has also been shown to significantly improve the recording characteristics of transmembrane action potentials, measured with nanovolcano arrays, which enabled intermittent intracellular access for up to 2 days.^236^

Nonetheless, the extent of PM electroporation can vary according to the local coupling and the amplitude of the electric field applied, making it very difficult to control the effective area of the cell–electrode interface that is going to indeed be affected. This may lead to cell damage and contact area degradation as visualized by means of focused ion beam scanning electron microscopy (FIB-SEM).^237^

PM poration and intracellular recording is also possible by means of laser optoacoustic poration^237^ through the ejection of electrons from the electrode tip upon laser exposure at a certain wavelength,^237,238^ as shown in Figure 10C. This approach indeed offers a controlled PM poration at the extremity of pseudo-3D electrodes of different size and geometry.^237^ Importantly, the localized effect of optoporation does not largely damage the cell PM, preserving the tight contact with the electrode and, compared to electroporation, enables significantly more stable and longer high-quality recordings, lasting 50 min on average.^237^

Although spontaneous PM penetration of high aspect ratio nanostructures has indeed been used for biocargo delivery,^217−220^ optoporation and electroporation can be used to secure intracellular access.^239^ However, even if the intracellular milieu is successfully contacted, this could not be the only route for biocargoes delivery. In fact, the topography-induced recruitment of the endocytic machinery might further contribute to such mechanism.^188^ Interestingly, electroporation through pseudo-3D hollow nanoelectrodes has also been used for the electrophoretic intracellular delivery of nanorods, providing a new method for on-chip accurate analysis of intracellular content on a single-cell level.^240^

### 3.5 Pseudo-3D Bio-interfaces with Conjugated Polymers

Recently, significant efforts were devoted in engineering CPs with pseudo-3D structures to get organic bio-electronics platforms able to combine topographical cues with the intrinsic properties of CPs. Taking advantage of the material composition, CPs can be patterned at the micro- and nanoscales as similarly achieved with other polymer-based materials.^241,242^ In this scenario, diverse patterning approaches (i.e., 3D additive manufacturing,^243,244^ selective etching,^245^ femtosecond laser patterning,^176^ and replica molding^246^) have been proposed to enhance the cell–chip coupling, aiming to create out-of-plane topographies to effectively pin cells at the PM domain.

For instance, the topography-driven PM deformation at the cell–material interface with PEDOT:PSS microgrooves or PEDOT:PSS-coated quartz nanopillars (Figure 7D,E) drastically reduced the cleft distance as visualized by means of FIB-SEM.^29,176^ Furthermore, the cell PM, adapting to the pseudo-3D features of the grooved substrate, made close contact and extended inside the patterned microstructure of the PEDOT:PSS substrate.^29,176^ The enhanced physical attachment was also confirmed by the increased cell layer impedance on the microgrooves measured by means of EIS.^176^

Similarly, a microwrinkled PEDOT:PSS film, designed to recapitulate the native ECM topographical anisotropies, improved cell adhesion: cells were able to spread across multiple ridges and showed an elongated morphology.^247^ Here, topography-driven contact guidance and cell alignment to the microwrinkles were also used to promote SH-SY5Y cells differentiation and neurite outgrowth,^248^ as well as C2C12 alignment and myotube maturation,^247^ highlighting the central role played by anisotropic topographical cues in guiding the development of oriented cytoskeletal structures.

**Figure 11:**
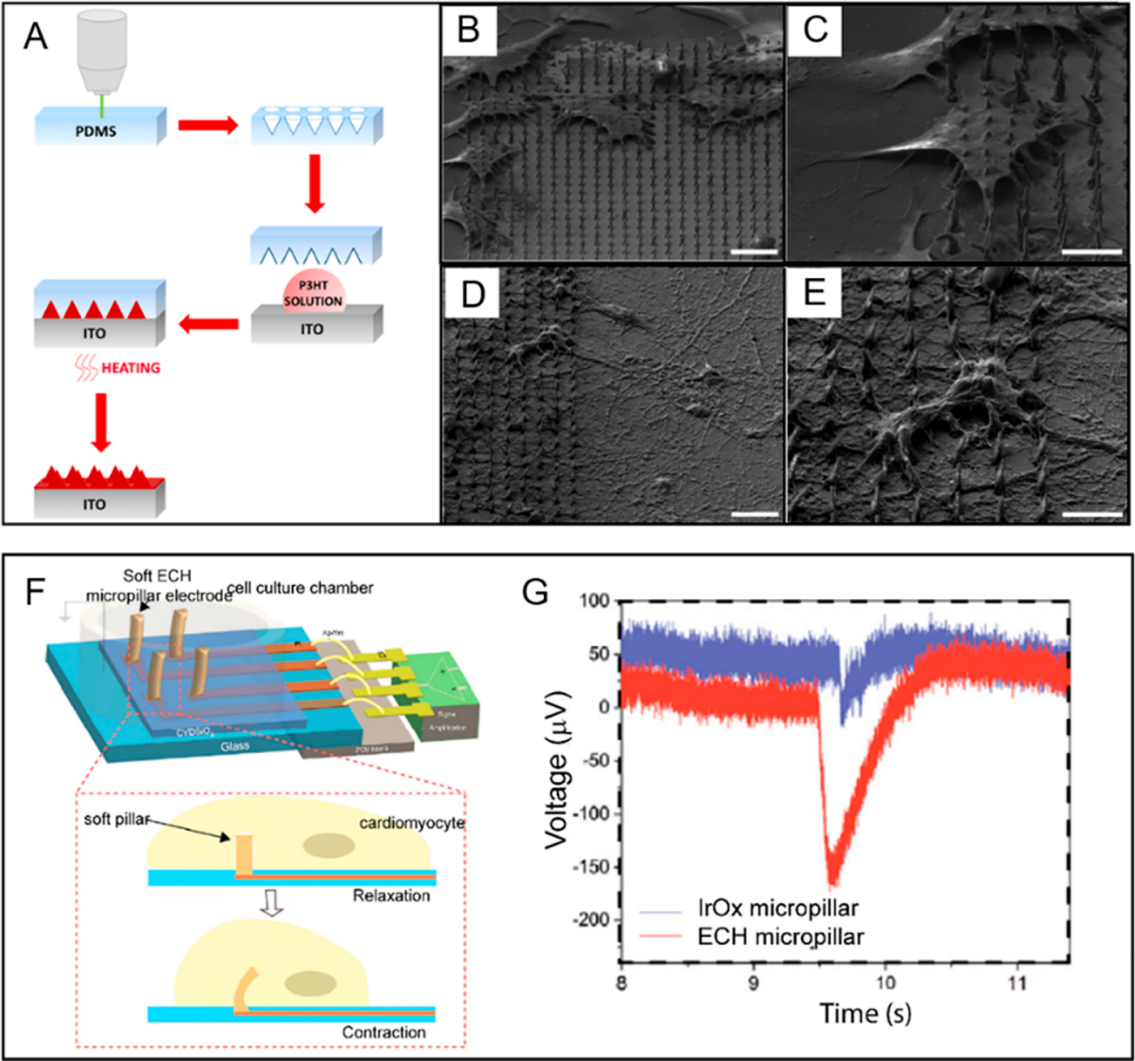
Pseudo-3D CP-based bio-interfaces. (A) Schematic representation of the P3HT micropillar fabrication process. (B) Scanning electron micrographs showing cells cultured on P3HT flat and microstructured substrates. Top-view SEM images of (C, D) HEK-293 cells and (D, E) cortical neurons cultured on the P3HT substrates. (F) Schematic representation of the soft hydrogel micropillar electrodes used for electrophysiological recording from cardiomyocytes (G) Extracellular recording of cardiomyocytes with IrOx micropillars and soft hydrogel micropillars. Reprinted and adapted with permission from the following: Ref (249). Copyright 2019 American Chemical Society. Ref (251). Copyright 2018 The Authors under Creative Commons International 4.0 Attribution license, published by National Academy of Sciences.

Recently, Tullii et al. fabricated P3HT polymer pillars (Figure 11A) whose conical shape allowed combination of the soft nature of the conductive polymer with the sub-micrometer rounded tip of the cone in order to establish a tight contact with cells (Figure 11B–-E). Indeed, the topography-mediated engagement of the PM, confirmed by a significant increase in membrane capacitance due to its thinning at the pillar tip, was able to trigger the activation of the cell cytoskeletal machinery and led to a substantial morphology remodeling of interfacing HEK-293 cells.^249^

However, despite the soft intrinsic nature of conductive polymers, the mechanical discrepancy at the cell–electrode interface in the aforementioned examples may still be such that the ability of the cell to effectively interact with a substrate might be hindered.

In order to further reduce the mechanical mismatch down to Young’s modulus values better recapitulating those found in native tissues,^250^ Liu et al. fabricated a soft micropillar electrode array made of a PEDOT:PSS hydrogel shown in Figure 11F: the significant reduction of the elastic modulus to tens of kPa promoted the formation of a tight seal at the interface as revealed by the electrode increased impedance measured by means of EIS.^251^ This finding was further confirmed by the large amplitude of the recorded extracellular HL-1 cell action potentials (Figure 11G), underlying how the soft nature of the micropillar electrode used was able to improve the electric coupling and be sensitive to the cell contraction.^251^

Finally, the application of an external voltage and the resulting volumetric expansion caused by the injections of ions into the polymer matrix can be used to mechanically stimulate cells and to engage the PM-mediated mechanotransduction machinery.

For instance, the actomyosin-mediated PM stretch induced by the mechanical stimulation of expanded PPy stripes was shown to increase the intracellular levels of Ca^2+^, probably due to the opening of mechanosensitive channels.^252^ The electrochemical switching of PPy can also be used to dynamically regulate attachment and detachment of mesenchymal stem cells: the combination of the volumetric expansion and the change in surface properties of the conductive polymer led to the switch between highly adhesive hydrophobic nanotubes to poorly adhesive hydrophilic nanotips, where the polymer expansion caused by voltage application reduced the inner diameter of the structure. The topography-driven activation of the cell mechanotransduction at the cell–material interface was confirmed by fluorescent microscopy and SEM, revealing focal adhesion formation and filopodia engaging with the tips and sides of nanotubes, but not with nanotips.^253^

## 4 Tissue-Electronics Bio-interfaces

### 4.1 Challenges in Engineering 3D Bio-electronic Interfaces

Because cells reside within the complex 3D environment of the ECM, further exploitation of its 3D features has to be considered when tailoring biomimicry strategies aimed at improving bio-interfaces. This is particularly relevant in tissue electronics, where the improved coupling between cells and 3D scaffolds^254^ is essential in triggering desired cellular responses at the cell–material interface^255,256^ and in enabling real-time electrical recordings.^257−260^ Moreover, further manipulation over cellular behavior may be required in order to prevent cell migration outside the scaffold, loss of cellular function and uncontrollable differentiation.^261−263^ In this regard, electrical stimulation has emerged as a novel tool to control this behavior in order to evoke specific cellular responses,^264^ especially for electrically excitable tissues such as bone, skeletal muscle, neural, and cardiac tissue.^265^

Tissue-like scaffolds are commonly made of natural or synthetic biodegradable polymers (e.g., polylactic acid (PLA), polycaprolactone (PCL), gelatin, and chitosan, etc.) that are electrically resistant.^15,265^ To enhance the electrical properties of such devices, inorganic conductive materials such as carbon nanotubes, graphene, or gold, can be employed,^266−273^ despite their non-biodegradability and the inhomogeneous distribution of the conducting elements.^265^

In this scenario, the soft nature of CPs, their biocompatibility, and electrical properties enabled the fabrication of stretchable, injectable, and implantable devices that can guide cellular in-growth while withstanding the tensile stress of developing tissue, thus paving the way for novel sensing^257−260^ and stimulation^259^ applications in tissue electronics. For instance, 3D scaffolds with nanoelectrodes with subcellular dimensions and sub-millisecond time resolution have been engineered for real-time action potential recordings.^257−260^ At the same time, the application of a simultaneous multisite electrical stimulation can be used to synchronize cardiac cell contraction^259^ or induce the release of drugs deposited on designated electrodes.^258−260^

**Figure 12:**
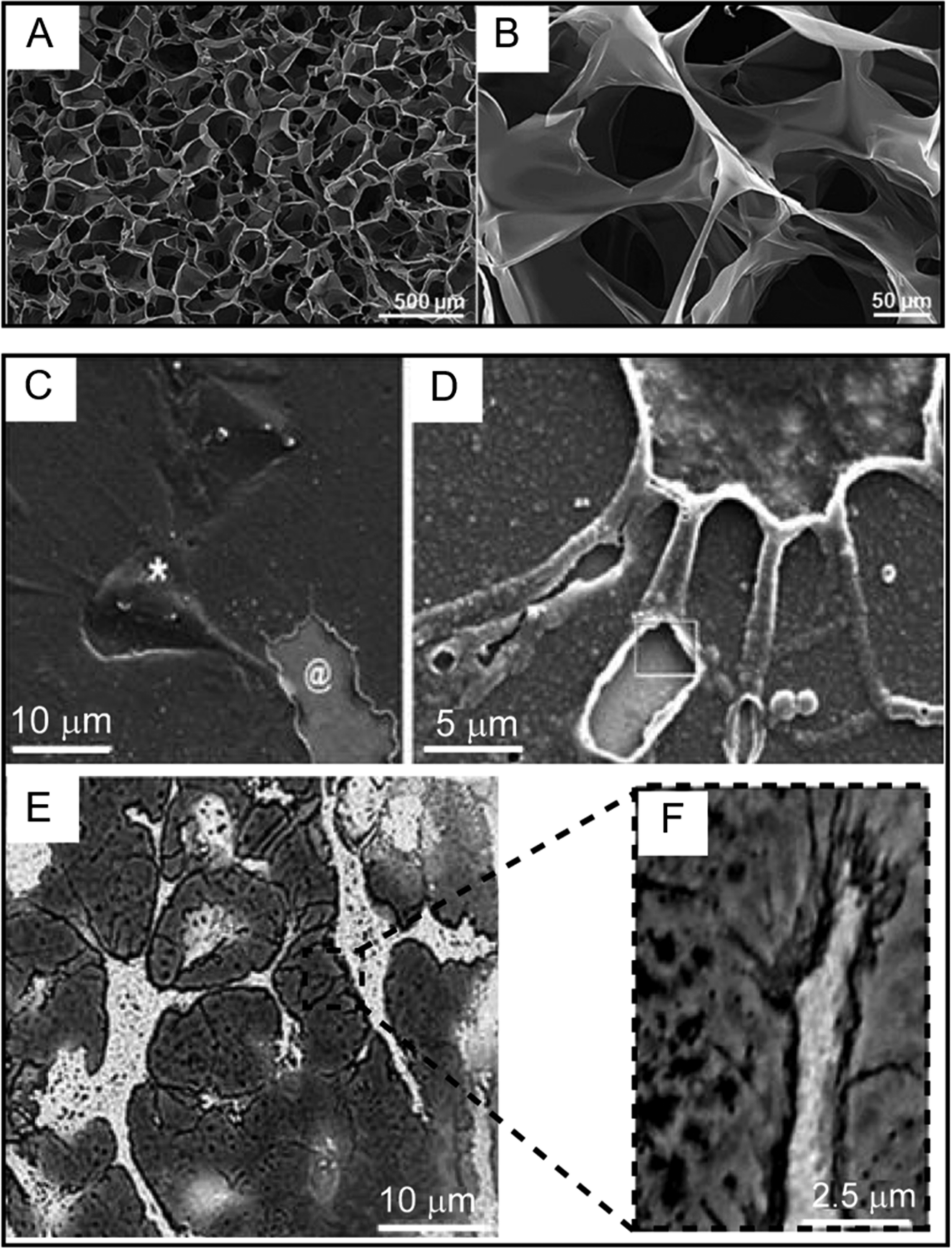
Engineering 3D bio-interfaces. (A and B) Scanning electron micrographs of highly porous ice-templated PEDOT:PSS scaffolds. (C) SEM image of neuron-templated PEDOT electrode coating revealing the cell-shaped holes. (D) Neurite-shaped tunnels and cracks created by the removal of the cells after PEDOT polymerization. (E) Optical images of neuron-templated PEDOT on Au/Pd electrodes. (F) Tight interface between PEDOT and cells enabling the visualization of micro- and nanofilopodia. Reprinted and adapted with permission from the following: Ref (284). Copyright 2017 Acta Materialia Inc. Published by Elsevier Ltd. Ref (288). Copyright 2006 Elsevier Ltd.

Despite their great potential, CPs are often brittle and non-biodegradable and possess limited solubility in organic solvents, requiring the development of innovative patterning techniques.^274−282^ For this reason, in order to achieve the fabrication of 3D-structured whole CPs, innovative approaches have been introduced. For instance, 3D macroporous scaffolds can be fabricated with PEDOT:PSS via an ice-templating method: here, the aqueous polymer dispersion is poured in a tube, and the subsequent sublimation of crystallized water returns a whole CP scaffold with a tubular geometry,^283−285^ as shown in Figure 12A,B. Pitsalidis et al. integrated a freeze-dried PEDOT:PSS scaffold into an electrochemical transistor configuration. Here, the 3D scaffold, supporting cell adhesion, and the tissue-mediated modulation of the transistor characteristics enabled the real-time monitoring of forming tissue-like architectures within the conducting polymer scaffold that constitutes the channel of the transistor.^285^

Conducting polymers can also be printed, cast, or synthesized in situ on pre-existing scaffolds by both oxidative polymerization and electrochemical deposition.^15,286,287^ For instance, PEDOT:PSS electrochemical deposition was used by Richardson-Burns et al. to achieve a neuron-templated 3D conducting polymer structure,^288^ as shown in Figure 12C,D. If this approach theoretically enables one to coat a great variety of 3D platforms already widely employed in tissue engineering, increasing exponentially the number of possible applications, it has the main drawback of limiting the conductivity only at the surface of the 3D-coated structure. In order to achieve biocompatible 3D-structured templates with bulk conductivity, CPs can be mixed with biodegradable polymers before being processed, ensuring efficient transmission of electrical signal throughout the whole composite.^289,290^

A substantial challenge when engineering 3D tissue-like platforms is that a trade-off must be sought between biodegradability and electronic functionality. An adequate proportion of electronically conducting subunits is necessary to achieve an appropriate conductivity, but the presence of nondegradable conducting units must be such that it can be tolerated and phagocytized without triggering any inflammatory response.^291−297^ In addition, the biopolymer–CP ratio can be tuned so that the degradation rate of a scaffold will match the maturation and regeneration of the forming tissue.^298^ For instance, the addition of PEDOT significantly enhanced the stability of a chitosan/gelatin scaffold to support adhesion of neuron-like PC-12 cells, delaying its biodegradation for up to 8 weeks,^291^ a time window normally necessary to allow neural tissue regeneration.^299^

### 4.2 3D Material Architectures Promote Integration and Electrical Coupling

Tissue-like platforms, acting as temporary template structures, should recapitulate the architectural properties of the ECM. Therefore, a controllable geometry, adequate porosity of suitable size, and an appropriate stiffness/elasticity are required to provide an ideal environment for cells to adhere and colonize the scaffold, facilitating in-growth of tissue and vasculature.

#### 4.2.1 Porosity

Substrate porosity, varying the distance between two adjacent anchoring points presented to adhering cells, plays an important role in mediating cell–substrate interactions.^300^ For instance, if the pore size exceeds the dimension of a cell, this will adhere to the surface of the scaffold as it would on a planar substrate.^250,301^ On the other hand, smaller pores of about the cell size, allow for the three-dimensional attachment, resulting in a different engagement of the mechanotranduction machinery.^250^ In this scenario, CP-based porous scaffolds have been widely employed in tissue engineering in order to emulate the 3D mesh-like structure of the ECM with an adequate porosity of suitable size in the range of 50–250 μm.^285,288,291,302^ The incorporation of CPs in polymer blends or its in situ polymerization on polymer-based scaffolds has also been shown to reduce scaffold pore size,^292,302^ creating a favorable environment for cells, while increasing the scaffold conductivity.

Furthermore, both in situ interfacial polymerization and incorporation in blend of CPs can also be used to increase surface roughness, contributing to observed beneficial effects of such platforms on cell adhesion and function.^291,293,303^

In order to obtain tissue-like scaffolds, Richardson-Burns et al. used in vitro polymerization of PEDOT in the presence of living neural cells to form a biomimetic conductive substrate with cell-shaped and cell-sized features, as shown in Figure 12C,D. The conducting polymer, molded around cell bodies and neurites, tightly adhered to the cell PM and exhibited nanoscale tendrils at the leading edge of extending neurites as visualized by means of SEM, as shown in Figure 12E,F. The combination of the nanometer-scale fuzziness and the cell-sized holes was shown to promote SH-SY5Y cells adhesion compared to untemplated PEDOT. Importantly, electrodes coated with neuron-templated PEDOT showed significantly enhanced electrical properties compared to bare electrodes, highlighting the potential of this innovative approach in the fabrication of a perfectly biomimetic microelectrode neural prosthetic able to mechanically assist and support neural network formation.^288^

Another strategy to combine the 3D architectural features of the ECM and the conductive properties of CPs uses PPy polymerized within a cardiogel. The cardiogel, a biosynthetic matrix obtained through the isolation of the ECM of cardiac tissue from its inhabiting cells (i.e., decellularization), provided a scaffold with a suitable pore size to support high density retention, infiltration, and colonization of cultured neonatal mouse cardiomyocytes. Furthermore, the PPy blend with the cardiogel in the presence of FeCl_3_ as doping agent resulted in an increase in electrical conductivity.^302^

**Figure 13:**
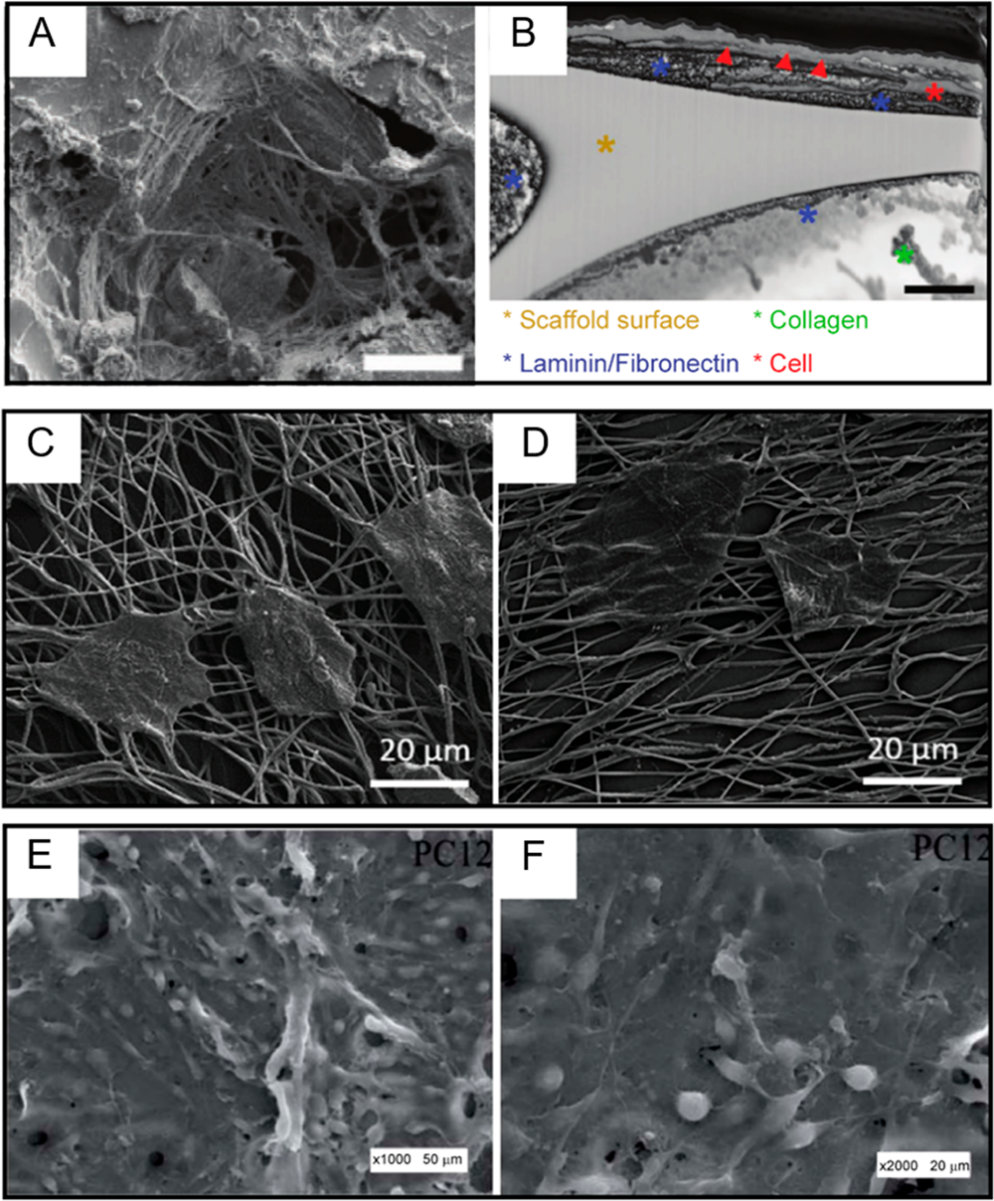
3D CP-based platforms to material promoting integration and electrical coupling. (A) Scanning electron micrograph of cells on porous PEDOT scaffolds (scale bar: 25 μm). (B) FIB-SEM image of a adipose cell revealing cellular and extracellular components of the 3D bio-interface (scale bar: 1 μm). (C and D) Scanning electron micrographs showing the effects of aligned PPy nanofibers on SHSY5Y cells. (E and F) Scanning electron micrographs of PC-12 cells adhering on a conductive sodium alginate and carboxymethyl chitosan hydrogel doped with polypyrrole. Reprinted and adapted with permission from the following: Ref (254). Copyright 2018 Wiley-VCH. Ref (314). Copyright 2019 The Royal Society of Chemistry. Ref (294). Copyright 2018 The Royal Society of Chemistry.

3D scaffolds with inductive niches of adequate porosity, promoting tight interactions between cells and the scaffold as shown in Figure 13A,B, can also trigger differentiation of cultured cells.^284,292,304^ This is particularly relevant in bone tissue formation, which is naturally guided by cross-linked collagen fibrils that act as a template onto which osteoblasts will deposit the mineralized bone matrix.^305^ Further details on scaffold materials, functionalization strategies, cell types, and applications are presented in Table 3.

**Table 3:** CP-Based 3D Biomimetic Scaffolds

material	functionalization type	cell type	application	ref
PCL/PEDOT	coating	human fetal mesenchymal stem cells	cytocompatibility	(306)
PLA coated with PANi	coating	bone marrow derived mesenchymal stem cells (BMSCs)	cell differentiation	(304)
PPy/chitosan	coating	cardiomyocytes	cell adhesion and maturation	(293)
PEDOT:PSS/gelatin/bioactive glass nanoparticles (BaG)	blend	adult human mesenchymal stem cells	cell adhesion and viability	(292)
PEDOT/Cs/Gel	blend	PC-12 cells	cell adhesion and proliferation	(291)
PEDOT:PSS	whole CP	MC3T3-E1 cells	cell differentiation	(284)
PEDOT:PSS	whole CP	MDCKII and TIF cells	cell adhesion and growth	(285)

#### 4.2.2 Geometry

Alignment patterns of tissues play a critical role in their functionality. For instance, for cardiac muscle cells to contract, cells and ECM need to be organized in such a way that they are elongated along the same axis. Another example of alignment is the skeletal muscle, where cells form long polynucleated units and are all elongated along the same axis.^5,256,307^

In order to dictate cell elongation and orientation, nanofibrous scaffolds can be fabricated by electrospinning to present cells with aligned contact guidance cues.^308^ Such structures, with fibers with an average diameter that ranges from 50 to 500 nm, have been widely employed to recapitulate the anisotropic architecture of the ECM. Furthermore, the incorporation of CPs before the electrospinning process has been shown to decrease fiber diameter,^303,309^ providing an increased surface area and more anchoring points for focal adhesion sites, underlying how the diameter reduction of the fibers becomes a tunable parameter to promote cell attachment. Moreover, the incorporation of CP nanoparticles before electrospinning can be used to obtain thin electrically conductive nanofibers, with increased surface roughness able to facilitate interaction with cells.^297,310^

In this scenario, composite nanofibers were used to promote myoblasts differentiation in mature, elongated, and multinucleated myotubes,^309,311^ highlighting how the combination of CPs mechanical and electrically conductive properties can direct skeletal muscle organization and tissue formation.

Similarly, PLA/polyaniline (PANi) nanofibers were shown to promote differentiation of H9c2 cardiomyoblasts, inducing the formation of gap junctions between cells which contributed to the formation of a functional beating cardiac network.^317^ Further details on nanofiber materials, functionalization strategies, cell types, and applications are presented in Table 4.

**Table 4:** CP-Based Nanofibers to Guide Cell Elongation and Orientation

material	functionalization type	cell type	application	electrical stimulation	ref
PPy-NPs/PCL	coating	MC3T3-E1 cells	cell adhesion and differentiation		(310)
PPy/PLC or PLGA	coating	PC-12 cells	cell differentiation		(312)
PPy/PCL	coating	DRG neurons	cell adhesion and differentiation	yes	(313)
PPy/cellulose	coating	SH-SY5Y cells	cell adhesion and differentiation	yes	(314)
P3HT/cellulose	coating	PC-12 cells	cell adhesion and differentiation	yes	(315)
PANi/poly(l-lactide-*co*-epsilon-caprolactone) (PLCL)	blend	C2C12 myoblasts	cell differentiation		(309)
gelatin/PANi/camphorsulfonic acid (CSA)	blend	C2C12 myoblasts	cell differentiation		(311)
PEDOT:PSS/chitosan	blend	rat bone marrow mesenchymal stem cell	cell adhesion and proliferation		(303)
PPy/PCL/chitosan	blend	PC-12 cells	cell adhesion and proliferation		(316)
PPy/chitosan/collagen	blend	human fibroblasts	cell adhesion and proliferation		(297)

The contact guidance cues provided by aligned nanofibers can also be used to guide neurite extension and promote differentiation in neuronal cells,^312,315,316^ as shown in Figure 13C,D, underlying the validity of such nanofibrous scaffolds as promising neural tissue substitutes. For instance, Wu et al. used P3HT micro- and nanofibers to guide cells’ neurite elongation and branches formation. Here, the photoconductive effect of P3HT upon light irradiation was exploited to provide electrical stimulation to cultured cells, offering further control over neuronal differentiation and directed growth.^318^

In addition, electrical stimulation can also be used to change the redox state of the organic CP and in turn modulate cell response. For instance, Ritzau-Reid et al. cultured neural stem cells on oligo-3,4-ethylenedioxythiophene (EDOT)-PCL fibers and observed enhanced neurite extension and branching when cells are exposed to electrical pulsing: this cellular response was attributed either to alterations in cell surface receptors and growth cone morphology or to changes in the redox states of the EDOT oligomer, which modifies the surface tension of the polymer.^319^

#### 4.2.3 Stiffness

The mechanical properties of a cell niche can vary between tissues, ranging from 0.1 kPa of soft brain tissue to 430 KPa of calcified bone.^5,250^ Importantly, the ideal ECM mechanical properties for a given cellular behavior have been shown to correlate with the elastic Young’s modulus (*E*) of the relevant living tissue.^24^ This cell-type-specific responsiveness may indeed have important clinical implications, as the elasticity of a 3D scaffold can be effectively adjusted in order to specifically guide cells as they mature and assemble into a certain tissue.^24,320^ For this reason, stiffer scaffolds are more frequently used to engineer bone-like structures,^284,292,321^ whereas hydrogels, with their reduced Young’s modulus, are more commonly used to recapitulate the mechanical properties of softer biological tissues.^294,322−325^ Combining the advantageous properties of hydrogels with the tunable properties of CPs enables the fabrication of biohybrid multifunctional materials in which the percentage of CPs can be carefully tuned to modulate hydrogel physical and mechanical properties, such as swelling, elasticity and degradation.

For instance, the inclusion of PEDOT:PSS in a gelatin methacryloyl significantly enhanced conductivity of the hydrogel, decreasing the swelling rate and the Young’s modulus. Furthermore, PEDOT:PSS significantly reduced the hydrogel impedance, enabling the excitation of abdominal muscle tissue via electrical stimulation.^323^

However, composite conductive hydrogels suffer from lower electrical conductivity because of the presence of the insulating nonconductive hydrogel network. For this reason, a lot of efforts have been devoted to the fabrication of whole conductive hydrogels. For instance, PPy can be easily synthesized in a hydrogel state in the presence of an oxidizing agent and a surfactant.^327^ Similarly, the simple addition of dimethyl sulfoxide (DMSO) to PEDOT:PSS, followed by dry-annealing and rehydration, leads to the formation of a patternable whole conductive hydrogel.^328^ Alternatively, increasing the ionic strength of the aqueous solution induces changes in the film morphology leading to the formation of conductive hydrogels. The so-formed hydrogels can be interconnected with a secondary polymer network to tune the mechanical properties without sacrificing the electrical conductivity.^329^

CPs can also be used to increase the surface roughness of hydrogels, providing more anchoring points for adhering cells.^295,296,323,324^ For instance, the incorporation of PEDOT:PSS in a collagen–alginate blend resulted in an electroconductive hydrogel with a fibrous microstructure with sand rose-like surface morphologies, improving neonatal rat cardiomyocytes’ cell adhesion and alignment. Furthermore, the increased electroconductivity promoted the physiological beating rate of cardiomyocytes while eliminating signs of internal arrhythmia.^330^

Because of hydrogels’ porosity and their high water uptake, the use of conventional photolithography processes to obtain a controlled micropatterning with sub-100 μm resolution is still a challenge. Recently PEDOT:PSS hydrogel has been successfully patterned in 3D surfaces through electrogelation. This technique takes advantage of the well-established methods of lithography to pattern polymers that form networks in the presence of ions.^331^ Alternatively, three-dimensional printing can be used to pattern CPs.^277,278,332^ Here, with addition of DMSO, the aqueous solution of commercial PEDOT:PSS can be converted into a viscous liquid, thus resulting in a conducting polymer ink which can be 3D printed with high resolution (over 30 μm). Additionally, the hydrogel grid can be used as a platform to culture dorsal root ganglion neurons, as shown by Heo et al., who demonstrated how increasing the percentage of PEDOT:PSS in the hydrogel influences cell adhesion and proliferation.^332^

The biomimetic potential of hydrogels can be further improved by biofunctionalization with integrin binding proteins. For instance, Xu et al. used a PEDOT:poly(ethylene glycol):RGD hydrogel, showing that the presence of the RGD sequence significantly increased the number of adhering mesenchymal stromal cells. The RGD-mediated activation of the mechanotransduction machinery was also confirmed by the cell’s more stretched morphology and the activation of the cytoskeleton.^333^ Here, upon electrical stimulation cells started to show variation in morphology and this caused an increase in the electrical conductivity of the hydrogel, thus showing how the formation of cell fiber bundles can influence the electron mobility in the 3D network. Similarly, electrical stimulation can be used to inducing the release of anti-inflammatory drugs, highlighting the potential of these materials in local therapy strategies.^333^ Further details on hydrogel materials, functionalization strategies, cell types, and applications are presented in Table 5.

**Table 5:** CP-Based Hydrogels

material	functionalization type	cell type	application	electrical stimulation	ref
PEDOT/carboxymethyl chitosan (CMCS)	coating	PC-12 cells	cell adhesion and proliferation		(295)
PANi/*N*-fluorenylmethoxycarbonyldiphenylalanine (Fmoc-FF)	blend	cardiomyocytes	cytotoxicity		(296)
PPy/collagen	blend	PC-12 cells	cell adhesion and differentiation	yes	(324)
PPy/sodium alginate (SA)/carboxymethyl chitosan (CMCS)	blend	PC-12 cells	cell adhesion and differentiation	yes	(294)
f-PEDOT/acrylic acid/hydroxyethyl methacrylate	blend	cardiomyocytes	cell adhesion		(325)
PPy/chitosan	blend	neonatal rat cardiomyocytes	citotoxicity and contraction		(326)

## 5 Conclusions and Future Perspectives

The fluid interface of the PM with its highly dynamic nature allows cells to respond and adapt to their surrounding environment, harnessing and transducing the informative cues provided the ECM. In this review we highlighted how the cellular mechanism underlying substrate tethering and cell adhesion can be exploited by biomimicry strategies tailored to effectively improve the cell–chip communication.

In particular, we underlined how the shift from planar to pseudo-3D electroactive substrates and the possibility of exploiting cells’ ability to respond to local changes in topography has significantly improved the electrical coupling, leading to high-quality electrophysiological recordings, biomolecules sensing, and stimulation. Here, the intrinsic soft nature of CPs can be further exploited to reduce the mechanical discrepancy at the cell–electrode interface, providing a mechanical environment in terms of Young’s modulus similar to that found in native tissues.

Because cells reside within the complex 3D environment of the ECM, further exploitation of its architectural features with conductive 3D tissue-like platforms has been instrumental in providing an ad hoc environment for in-cell growth, while enabling stimulation and sensing applications.

Additionally, the interface with cells can be further tightened by exploiting biofunctionalization strategies, based on ECM-derived proteins and growth factors. In this scenario, we highlighted how this approach may pave the way for the use of artificial membranes as functional coatings of electronic devices, leading to the development of a new class of biosensors able to study biological events in real time.

In conclusion, in this work we untangled how the complex mechanisms underlying PM-mediated interactions with CP-based platforms can be exploited in tailoring specific engineering strategies aimed at fabrication of electronic devices in order to promote their seamless integration with the biological world.

## List of Abbreviations

BAR: Bin/Amphiphysin/Rvs
CP: conductive polymers
DMSO: dimethyl sulfoxide
DPP3T: dithienyldiketopyrrolopyrrole and thiophene
DRG: dorsal root ganglion
ECM: extracellular matrix
EDL: electrical double layer
EDOT: 3,4-ethylenedioxythiophene
EIS: electrochemical impedance spectroscopy
FET: field-effect transistor
FIB-SEM: focused ion beam–scanning electron microscopy
HD-MEA: high-density multielectrode array
MEA: multielectrode array
NGF: nerve growth factor
OECT: organic electrochemical transistor
P3HT: poly(3-hexylthiophene)
PANi: polyaniline
PCL: polycaprolactone
PDLO: poly-dl-ornithine
PEDOT: poly(3,4-ethylenedioxythiophene)
PLA: poly(lactic acid)
PM: plasma membrane
PPy: polypyrrole
PSS: poly(styrenesulfonate)
pTS: *p*-toluenesulfonate
SALB: solvent-assisted lipid bilayer
SEM: scanning electron microscopy
SLB: supported lipid bilayer
TAT: trans-activating transcriptional activator
VF: vesicle fusion
